# Multi-Scale Light-Sheet Fluorescence Microscopy for Fast Whole Brain Imaging

**DOI:** 10.3389/fnana.2021.732464

**Published:** 2021-09-24

**Authors:** Zhouzhou Zhang, Xiao Yao, Xinxin Yin, Zhangcan Ding, Tianyi Huang, Yan Huo, Runan Ji, Hanchuan Peng, Zengcai V. Guo

**Affiliations:** ^1^School of Medicine, IDG/McGovern Institute for Brain Research, Tsinghua University, Beijing, China; ^2^School of Life Sciences, Tsinghua University, Beijing, China; ^3^Tsinghua-Peking Joint Center for Life Sciences, Beijing, China; ^4^SEU-Allen Joint Center, Institute for Brain and Intelligence, Southeast University, Nanjing, China; ^5^Allen Institute for Brain Science, Seattle, WA, United States

**Keywords:** light-sheet microscopy, whole brain imaging, multi-scale imaging, electrode localization, single neuron morphology

## Abstract

Whole-brain imaging has become an increasingly important approach to investigate neural structures, such as somata distribution, dendritic morphology, and axonal projection patterns. Different structures require whole-brain imaging at different resolutions. Thus, it is highly desirable to perform whole-brain imaging at multiple scales. Imaging a complete mammalian brain at synaptic resolution is especially challenging, as it requires continuous imaging from days to weeks because of the large number of voxels to sample, and it is difficult to acquire a constant quality of imaging because of light scattering during *in toto* imaging. Here, we reveal that light-sheet microscopy has a unique advantage over wide-field microscopy in multi-scale imaging because of its decoupling of illumination and detection. Based on this observation, we have developed a multi-scale light-sheet microscope that combines tiling of light-sheet, automatic zooming, periodic sectioning, and tissue expansion to achieve a constant quality of brain-wide imaging from cellular (3 μm × 3 μm × 8 μm) to sub-micron (0.3 μm × 0.3 μm × 1 μm) spatial resolution rapidly (all within a few hours). We demonstrated the strength of the system by testing it using mouse brains prepared using different clearing approaches. We were able to track electrode tracks as well as axonal projections at sub-micron resolution to trace the full morphology of single medial prefrontal cortex (mPFC) neurons that have remarkable diversity in long-range projections.

## Introduction

Imaging whole brains at cellular and subcellular resolutions plays an increasingly important role in deciphering the structure and function of neural circuits at the normal and abnormal states (Ahrens et al., 2013; Renier et al., 2014; Liebmann et al., 2016; Harris et al., 2019; Roostalu et al., 2019; Winnubst et al., 2019; Peng et al., 2020). Brain-wide imaging of sparsely labeled neurons at near sub-micron resolution in the *x*-*y*-*z* axis combined with single-neuron tracing demonstrates intricate arborization of individual neurons across brain areas (Winnubst et al., 2019; Peng et al., 2020). Current whole-brain micro-imaging methods, such as micro-optical sectioning tomography (MOST) and serial two-photon tomography, typically take about 1 to 2 weeks to image the whole mouse brain at ∼0.3 μm × 0.3 μm × 1 μm voxel and produce 10–20 TBs of data that are challenging to handle (Li et al., 2010; Gong et al., 2013; Economo et al., 2016). Meanwhile, there are applications that only require cellular or micrometer resolution (Ahrens et al., 2013; Renier et al., 2014; Harris et al., 2019), and imaging at sub-micrometer resolution not only slows down data acquisition speed but also produces a larger dataset that challenges the power of computation. Thus, it is highly desirable to image whole-brain at appropriate resolutions in order to increase the speed of data acquisition as well as to reduce the acquired dataset size, and accordingly, difficulty in handling big data. Although it is highly desirable, not every imaging method is suitable for multi-scale imaging.

**FIGURE 1:**
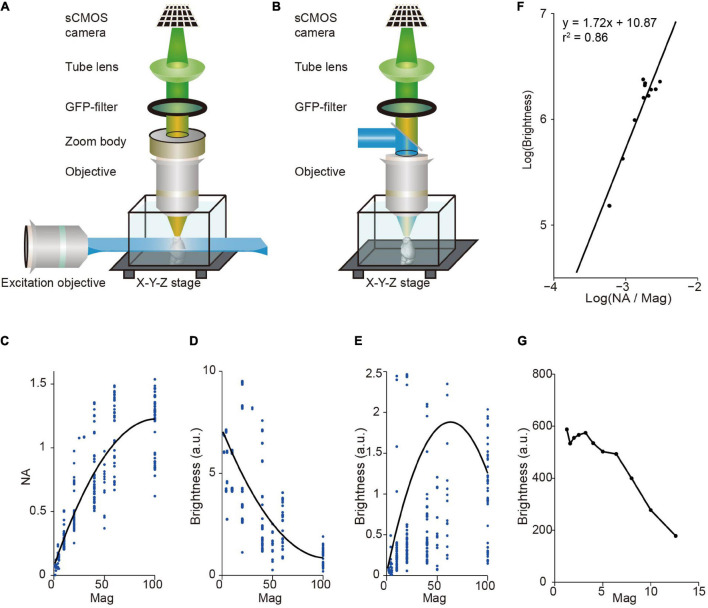
Image brightness in light sheet fluorescence microscopy (LSFM) and wide-field fluorescence microscopy. **(A)** LSFM is based on the principle of decoupling illumination and detection by generating a thin “sheet” of light from the side using a dedicated objective to induce optical sectioning. **(B)** Wide-field epi-fluorescence microscopy uses the same objective for specimen illumination and fluorescence detection. This approach couples illumination and detection. **(C)** Numerical aperture (NA), on average, increases monotonically with magnification for commercial objectives. Dots, individual objectives (Nikon and Olympus, downloaded from official websites). Black curve, fitted with a second-order polynomial function. **(D)** Brightness, on average, decreases monotonically with magnification for commercial objectives in LSFM. Same data as in **(C)**. Black curve, fitted with a second-order polynomial function. **(E)** Relationship between brightness and magnification for epi-fluorescence microscopy (same objectives as in **C,D**). Brightness is highest around 60× magnification, indicating that using oil immersion objectives with 40–60× magnification can achieve brightest images. Note that dotes above 2.5 are not shown. Black curve, fitted with a second-order polynomial function. **(F)** Measured signal brightness and NA/Magnification (Mag) (dots) by imaging a dye pool in LSFM. The brightness of signal is roughly proportional to the square of the numerical aperture, and to the inverse square of the magnification of the detection objective (i.e., brightness ∝ NA^2^/Mag^2^). **(G)** Measured signal brightness (dots) at different magnifications in LSFM (same data as in **F**).

Light-sheet fluorescence microscopy (LSFM) has a unique advantage in multi-scale imaging compared with wide-field fluorescence microscopy. The brightness of signal depends on the light-condensing power of the illumination objective (∝NA^2^), the light-gathering power of the collection objective (∝NA^2^), and the magnification of image (∝1/Mag^2^), where NA is the numerical aperture of the objective, and Mag is magnification. Light-sheet fluorescence microscopy (LSFM) decouples the illumination and detection optical paths by utilizing a thin plane of light that is perpendicular to the axis of the detection objective for excitation (Siedentopf and Zsigmondy, 1902; Huisken et al., 2004; Keller and Ahrens, 2015). Thus, in LSFM, image brightness is proportional to NA^2^/Mag^2^ (as the light-sheet thickness for illumination is typically fixed) whereas in wide-field fluorescence microscopy it is proportional to NA^4^/Mag^2^ (Figures 1A,B). For objectives with standard parfocal distance and typical field number from major manufactures, NA is positively correlated with Mag, but it increases slower at larger Mag (Figure 1C). Thus, in LSFM, image brightness is typically much higher when using low magnification objectives (Figure 1D). For comparison, in wide-field fluorescence microscopy, brightness is highest when using oil immersion objectives with 40–60× magnification (Figure 1E). Indeed, image brightness tested by imaging a dye pool in light-sheet configuration is roughly proportional to NA^2^/Mag^2^ with a brighter signal at low magnification (Figures 1F,G). This reveals a fundamental difference between LSFM and wide-field fluorescence microscopy, suggesting that LSFM is especially suitable for multi-scale imaging.

We focused on whole brain structural imaging by exploiting the unique advantage of LSFM in multi-scale imaging. Despite the huge success of LSFM, especially with advanced light-sheet imaging and tissue clearing techniques (Dodt et al., 2007; Erturk et al., 2012; Chung et al., 2013; Susaki et al., 2014; Tomer et al., 2014; Hama et al., 2015; Richardson and Lichtman, 2015; Pan et al., 2016; Narasimhan et al., 2017; Murakami et al., 2018; Yang et al., 2018; Hillman et al., 2019; Voigt et al., 2019; Wang et al., 2019; Chen et al., 2020; Ueda et al., 2020a,b; Xu et al., 2020; Zhu et al., 2020; Choquet et al., 2021), mammalian brains still present a huge challenge for *in toto* imaging because of light scattering and insufficient clearing of the central portion of the brain. Thus, LSFM techniques also combine with tissue sectioning to achieve a constant quality of imaging of mouse brains at a cellular resolution within a few hours (Narasimhan et al., 2017; Yang et al., 2018) and monkey brains within 100 h (Xu et al., 2020). However, due to the tradeoff among spatial resolutions, the field of view (FOV), acquisition time, and constant quality of imaging, the brain-wide imaging of mammalian brains typically has a dilemma of not being able to ensure high resolution (especially along the axial direction) and high-speed acquisition simultaneously.

Here we have developed multi-scale light sheet fluorescence microscopy (mLSFM) that achieves constant quality of brain-wide imaging from cellular (3 μm × 3 μm × 8 μm) to sub-micron (0.3 μm × 0.3 μm × 1 μm) spatial resolution rapidly within a few hours. Tiling light-sheet across the FOV with automatic sectioning to remove the imaged top section (when necessary) enables constant quality of imaging across the whole brain at synaptic resolution (i.e., the resolution to visualize axonal terminals and dendritic spines). Automatic optical zoom-in and adjustment of light-sheet thickness allow flexible change of magnification during imaging. By combining efficient signal detection at low magnification enabled by light-sheet but not wide-field imaging and high-resolution imaging of signals, mLSFM achieves whole brain imaging of sparsely labeled neurons at synaptic resolution at a speed more than 10 times faster than previous methods.

## Materials and Methods

### Animal Care and Use

Both male and female mice ≥8 weeks were used. The animals were housed in three to five groups and maintained on a 12-h light/dark cycle and at 22–26° with sterile pellet food and water *ad libitum*. All animal experimental procedures were conducted with approved protocols in accordance with Tsinghua University guidelines, and were approved by the Institutional Animal Care and Use Committee (IACUC) of Tsinghua University.

### Virus Injection and Sparse Neuronal Labeling

We used AAV2/1-hSyn-Cre and AAV2/1-CAG-Flex-EGFP viruses for sparse neuronal labeling and AAV2/1-CAG-tdTomato (UNC) for bulk injection. Both the AAV2/1 hSyn-Cre virus and the AAV2/1 CAG-Flex-EGFP virus were obtained from Addgene (Watertown, MA, United States): pENN-AAV-hSyn-Cre-WPRE-hGH was a gift from James M. Wilson (Addgene viral prep # 105553-AAV1^1^; RRID: Addgene_105553); AAV pCAG-FLEX-EGFP-WPRE was a gift from Hongkui Zeng (Addgene viral prep # 51502-AAV1^2^; RRID: Addgene_51502) (Oh et al., 2014). To achieve sparse labeling of a few dozens of cortical neurons, 50 nl of AAV2/1-hSyn-Cre (final titer ∼5 × 10^7^ GC/ml) and AAV2/1-CAG-Flex-EGFP (∼2 × 10^12^ GC/ml) was injected into mPFC (AP: +1.85, ML: 0.4, DV: 1.7) unilaterally at a rate of 17 nl/min. To achieve bulk labeling of population neurons, 70 nl of AAV2/1-CAG-tdTomato was injected into ALM (anterior lateral motor cortex, AP: +2.5, ML: 1.5, DV: 0.8) or mPFC (AP: +1.85, ML: 0.4, DV: 1.7) unilaterally at a rate of 50 nl/min. The injection procedure was the same as before (Guo et al., 2017). Briefly, the mice were kept anesthetized under 1–2% isoflurane during the whole injection procedure. The viral mixture was delivered using an oil hydraulic micromanipulator (MO-10; Narishige, Tokyo, Japan). For sparse labeling of mPFC neurons, the mice were maintained for 5 weeks to allow a stable and strong expression of EGFP in a few dozens of neurons. For bulk labeling of mPFC and ALM neurons, the mice were maintained for 4 weeks.

### Tissue Clearing and Sample Embedding for Imaging

#### CUBIC-X Clearing

Five weeks after virus injection, the mice were anesthetized with a 0.5% pentobarbital sodium solution (0.4 ml/30 g body weight), and transcardially perfused with 0.1 M phosphate-buffered saline (PBS) containing 20 U/ml heparin. The internal liquid pressure during perfusion was adjusted to be low (∼70 mmHg) during the first 10 min of perfusion, followed by another 10 min at a higher pressure (∼100 mmHg). We found that the higher pressure potentially reduced autofluorescence from blood vessels that interfered with the detection of neuronal signals during imaging. After PBS, the mice were perfused with 4% paraformaldehyde (PFA) in 0.1 M PB.

Brains were dissected and post-fixed in 4% paraformaldehyde for 2 days at 4°. After washing with PBS for 1 day with the solution changed at 6 and 12 h, the brain samples were delipidated with a CUBIC-1 solution for 6 days at room temperature (the solution was changed on days 3 and 5) (Murakami et al., 2018). Following washing in PBS for 1.5 days with solution change at 6, 12, and 24 h, the brain samples were immersed in a CUBIC-X1 swelling solution for 3 days at 4°C (the solution was changed daily). Finally, the expanded brain was moved to refractive index matching solution CUBIC-X2 for 3 days with solution change every day.

After refractive index matching, the brain samples were embedded in 4% agarose dissolved in the CUBIC-X2 solution (without imidazole component for better adherence), and then were glued on the sample stage using an instant adhesive (Loctite 401, Henkel, Shandong, China).

#### CUBIC-MACS Clearing

The brain samples were delipidated with the CUBIC-1 solution for 16 days at 4°C (solution was changed every 4 days). After washing in PBS for 1.5 days, the brain samples were serially incubated in 50 ml MACS-R0 and MACS-R1 solutions with gentle shaking at room temperature (Zhu et al., 2020). The brain samples were incubated in MACS-R0 for 1.5 days, and in MACS-R1 for 0.5–1 day until they became transparent.

#### uDISCO Clearing

The mice were deeply anesthetized with a 0.5% pentobarbital sodium solution (0.4 ml/30 g body weight). Then, the mice were transcardially perfused with 0.01 M PBS (Sigma-Aldrich Inc., St. Louis, MO, United States) to flush blood vessels, and with 4% paraformaldehyde (PFA, Sigma-Aldrich Inc., St. Louis, MO, United States) in PBS (pH 7.4) for fixation. The brain samples were post-fixated with the same fixation solution at 4°C for 24 h. After fixation, each intact brain was rinsed with PBS for several hours to remove PFA. The ultimate 3D imaging of solvent-cleared organs (uDISCO) dehydration procedure consisted of serial incubation in 30 ml of 30, 50, 70, 80, 90, 96, and 100 vol% tert-butanol at 37°C water bath (Pan et al., 2016). Then, the brain samples were immersed in 30 ml of dichloromethane (DCM) for 2 h at room temperature; finally, they were transferred to solution of benzyl alcohol-benzyl benzoate and diphenyl ether mixed at a volume ratio of 4:1 (BABB-D4) at 37°C water bath until they became transparent.

#### MAP Clearing

The expansion of mouse tissue with magnified analysis of the proteome (MAP) was performed as previously described (Ku et al., 2016). After being anesthetized with a 0.5% pentobarbital sodium solution, the mice were perfused with a mixture of 5% acrylamide (AA), 0.05% bis-acrylamide (BA), and 0.8% sodium acrylate (SA) in PBS, and then perfused with the fixative solution containing a mixture of 4% PFA, 30% AA, 0.1% BA, 10% SA, and 0.1% VA-044 in PBS. The brain samples were dissected out and incubated in the fixative solution at 4°C overnight. After PBS washing, the brain samples were embedded in 2% agarose gel and were sectioned into 1-mm coronal slices using a vibratome (VT1200s; Leica, Wetzlar, Germany). The brain slices were then incubated in the fixative solution at room temperature for 1 day, with gentle shaking, followed by incubation for 2 h at 45°C for hydrogel tissue hybridization. After hybridization, the hydrogel was cleared by incubating the brain slices in a solution of 200 mM SDS, 200 mM NaCl, and 50 mM Tris in DI water (pH titrated to 9) at 37°C with gentle shaking. Finally, the brain slices were immersed in ddH_2_O overnight to allow for expansion.

### Microscope Setup

**FIGURE 2:**
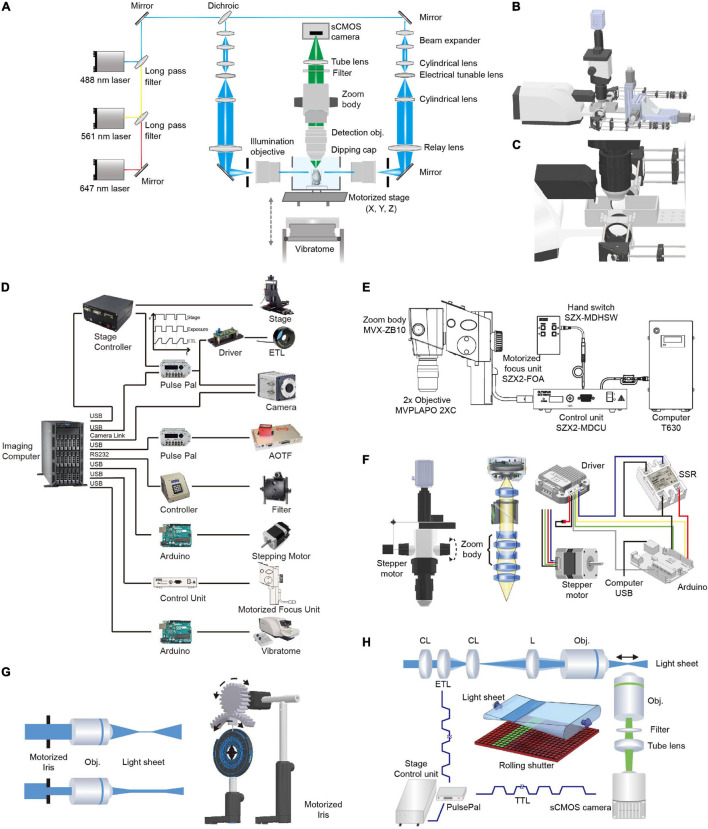
Optical layout and hardware control in multi-scale LSFM. **(A)** Optical layout of the customized LSFM. **(B)** Inventor three-dimensional (3D) rendering of the customized LSFM. **(C)** Zoomed-in view of the imaging chamber, detection objective, illumination objectives, and the vibratome. The detection objective, Olympus MVPLAPO 2X, has an NA of 0.5 and a working distance (WD) of 20 mm. As the detection objective is an air objective and the imaging media, such as benzyl alcohol-benzyl benzoate (BABB), has a refractive index of 1.56, a 3D-printed dipping cap was attached to the objective to reduce spherical aberration. The two illumination objectives, Olympus MVPLAPO 1X, have an NA of 0.25 and a WD of 65 mm that leaves enough space for the imaging chamber. **(D)** Hardware control diagram. Mechanical stages, AOTF (shutter and laser intensity control), filter wheel, stepping motor, motorized focus unit, and vibratome are directly controlled with a computer through their controllers. To synchronize the electrically tunable lens (ETL) with virtual slit detection, the Z-stage at specified positions sends out a transistor–transistor logic (TTL) signal to trigger camera exposure and ETL focus. **(E)** Adjustment of objective focus. The motorized unit (SZX2-FOA; Olympus, Tokyo, Japan) with a control unit (SZX2-MDCU) was used to refocus between different zoom settings, as there is a small focus difference at different zoom magnifications. **(F)** Adjustment of magnification. A stepper motor (UI robot) was attached to the shaft of the magnification knob to automatically adjust magnification. The stepper motor was computer-controlled *via* its driver and an Arduino board (UNO). An solid state relay (SSR) was used to switch between the manual and automatic adjustments of magnification. **(G)** Light-sheet thickness was adjusted using a motorized iris to trim the beam diameter entering the illumination objective. The motorized iris was customized by attaching a stepper motor (UI robot) to an iris (ID50/M; Thorlabs, Newton, NJ, United States) through a pair of three dimensional (3D)-printed gears. **(H)** Synchronization between ETL focus and camera exposure during non-stop Z scanning. The customized linear stage continuously moves along the *Z* axis and sends out a TTL signal every 2 μm to synchronize ETL focus and camera exposure.

The optical layout and control diagram are shown in Figure 2. Briefly, 488- (OBIS 488-100 LS; Coherent, Santa Clara, California, United States), 561- (OBIS 561-100 LS; Coherent), and 640-nm (OBIS 640-100 LS; Coherent) lasers were combined and then 50:50 split into two paths for dual-sided illumination. For each illumination path, the light exiting from an optical fiber was collimated with an adjustable aspheric collimator (CFC-11X-A; Thorlabs, Newton, NJ, United States) and then expanded five times using two achromatic lenses (ACN254-040-A, AC254-200-A; Thorlabs, Newton, NJ, United States). The expanded parallel light beam then passed through a telescope consisting of two cylindrical lenses (LJ1810L2-A, *f* = 24.88 mm; LJ1703RM-A, *f* = 75 mm; Thorlabs, Newton, NJ, United States) with an electrically tunable lens (ETL, EL-10-30-VIS-LD; Edmund Optics, Barrington, NJ, United States) positioned at the focal length of the first cylindrical lens. Thus, the light beam exiting from the telescope was parallel along one axis (the horizontal axis in the setup) independent of the focal length of the ETL (as the ETL was positioned at the focal length, Figure 2H). Along the perpendicular vertical axis, the focal point of the light beam passing through the ETL could be modulated by changing the voltage applied to the ETL (a constant voltage was applied to maintain the focal length at 75 mm and deviation from the voltage changed the focal length from 75 mm) (Hedde and Gratton, 2016). Then, the beam was focused into the back aperture of the illumination objective (MVPLAPO 1X, WD = 65 mm, diameter, ∼65 mm; Olympus, Tokyo, Japan) with a relay lens (AC508-200-A, *f* = 200 mm, Thorlabs, Newton, NJ, United States). As the objective had a large aperture, the beam was expanded ∼3× along the vertical axis to largely fill the back aperture of the illumination objective. The fraction of back aperture illuminated and thus, the thickness of the light-sheet at the sample were automatically controlled by a custom-made electric diaphragm. To facilitate the alignment of the light-sheets to the detection plane, optics after the optical fiber were mounted on a 3D stage (MTS204; Beiguang Shiji, Beijing, China).

The detection light path is orthogonal to the illumination path. To flexibly change the magnification, we employed a macro-scope zoom body (MVX10; Olympus, Tokyo, Japan) coupled with an MVPLAPO 2x (Olympus, Tokyo, Japan) (inspired by the design of ultramicroscope) (Dodt et al., 2007). To automatically change the zoom ratio (from 0.63× to 6.3×), we attached a stepping motor to the zoom wheel and used an Arduino board (Arduino, Boston, MA, United States) to generate pulses to drive the motor. As the focal plane slightly drifts between different zoom settings, we used the Olympus motorized focus unit (SZX2-FOA; Olympus, Tokyo, Japan) combined with its control unit (SZX2-MDCU; Olympus, Tokyo, Japan) to correct the difference in focal planes. To make the air objective compatible with various clearing techniques such as the hydrophilic reagent-based method CUBIC-X (Murakami et al., 2018) and organic solvent approach uDISCO (Pan et al., 2016), we customized an aberration correction adapter and a waterproof dipping cap for the MVX-10 2× objective. After the emission light exited the zoom body, it passed through a filter (59022m, ET655lp, or ZET405/488/561/640m; Chroma Technology Corp., Bellows Falls, VT, United States) installed in a filter wheel (Lambda 10-B; Sutter Instrument, Novato, CA, United States). To project light to the camera chip, a tube lens (MVX-TLU; Olympus, Tokyo, Japan) and a C-mount adapter (MVX-TV1xC; Olympus, Tokyo, Japan) were attached to a scientific complementary metal–oxide–semiconductor (sCMOS) camera (Orca Flash 4.0 V2; Hamamatsu Photonics, Shizuoka, Japan). During imaging, a Z-stage-initiated trigger signal synchronized the rolling shutter (“light-sheet mode”) with the tunable lenses to enable uniform axial resolution across a large field of view.

### Image Acquisition

Control of devices and data acquisition were performed using a computer equipped with an Intel Xeon E5-2630 v.4 processor operating at 2.2 GHz, 80 GB of 2133 MHz DDR4 memory, and an integrated Inter AHCI chipset controlling 14 × 4 TB 7.2K RPM hard disks in a RAID0 configuration. Scripts written in MATLAB (version 2018a; MathWorks, Natick, MA, United States) handled the scanning, stage motion, vibratome cutting, and image data acquisition. Laser power and on-off profile were controlled *via* an acousto-optic tunable filter (AOTF) (AA Opto-Electronic, Orsay, France) and a programmable pulse train generator (Pulse Pal Gen2; Sanworks, Rochester, NY, United States). Tissue sections were cut with a vibratome (VT1200; Leica, Wetzlar, Germany), which was controlled *via* an Arduino board. As the brain samples were cleared, cutting was required every 1–5 mm, depending on the degree of clarity and imaging requirement. To facilitate the imaging of large brain samples of 1–2 cm, we chose linear translation stages with travel range of 100 mm × 50 mm × 50 mm (X/Y/Z; Hogan Instruments, Beijing, China). The customized linear stage for the *Z*-axis had a high-speed scanning mode: the stage moved continuously (typically at 0.1 mm/s) and sent a transistor–transistor logic (TTL) pulse every 1–5 μm (delay time <5 ms) to synchronize the exposure of the sCMOS camera and the driving voltage to the ETL.

Whole-brain imaging was performed by automatically repeating the following steps until the brain was imaged completely: (i) the X-Y-Z stages moved the sample under the objective, and a number of optical Z stacks were imaged as mosaic of fields of view, with 5% overlap in the X-Y plane; (ii) at the end of mosaic optical sectioning, the Z stage moved the sample upward, with 10% overlap in the Z direction between optical sections. When necessary, the sample was directed toward the vibratome by the X-Y stage, and the imaged section (minus the overlap region) was sectioned off.

Multi-scale whole brain imaging for sparsely labeled samples was performed by repeating the following steps: first, we performed overview scans with the lowest magnification (zoom body was set to 0.6×) to determine the boundary of the brain at the focus plane. Then, the section was divided into overlapping subregions to be imaged for signal detection. Second, all the subregions were scanned at 1.6× magnification. The images were acquired with 50 ms exposure time and with 20 μm Z-spacing. Then, the Z-MIP image of each tile was divided into 4 × 4 overlapping subregions, and each subregion was analyzed by a custom written script in MATLAB (MathWorks, Natick, MA, United States) to detect fluorescent signals. Briefly, the background (mean averaged with a rolling ball region) was subtracted. Images with intensity above a high threshold (typically 2× standard deviation above background) were classified with signals. Images with intensity below a low threshold (typically 0.5× standard deviation above background) were automatically classified as without signals, and the rest of the images were manually inspected for signals. Third, all tiles with neuronal signals were scanned with highest magnification (zoom body was set to 6.3×). During acquisition, the Z motor stage moved at 0.08 mm/s and generated a TTL signal every 2 μm to trigger the synchronization of camera and ETL. Finally, the imaged section was trimmed off using a vibratome to leave an overlap slice of 100 μm (10%) between sections.

### Characterization of Resolution With Fluorescent Beads

Fluorescent beads of 0.2 μm diameter were imaged to characterize the resolution of the system. The beads were embedded in a 2% agarose gel block that was placed in a 3D printed chamber with water bath. The excitation wavelength was 488 nm, and the corresponding emission filter was a bandpass filter (59022m; Chroma Technology Corp., Bellows Falls, VT, United States). The pixel size of the detection system was 0.52 μm under the magnification of 12.6× given the camera pixel size of 6.5 μm. Regions containing single beads were cropped out, and then the point spread function (PSF) was generated using the MetroloJ plugin in Fiji. The experimentally observed PSF from the beads was 0.95 μm at full-width half-maximum (FWHM), which was smaller than the theoretical resolution of 0.63 μm (calculated using the objective NA of 0.5). Thus, the measured PSF was limited by twice of the pixel size, resulting in an under-estimated resolution of the optics of the system. To characterize the PSF along the *z* axis, we moved the sample at a step size of 1 μm, and the measured PSF along Z was 2.1 μm, about twice of the step size, suggesting that the axial resolution is also limited by the step size of the motor, and the thickness of the light-sheet was, at most, 2 μm.

### Reconstruction of Recording Locations

The procedure of extracellular recording is the same as before (Guo et al., 2014; Huo et al., 2020). Briefly, a 64-channel silicon probe (four shank probes with 250-μm shank spacing and 25-μm site spacing; Diagnostic Biochips, Glen Burnie, MD, United States) was inserted at an oblique angle (∼45°) to reach the thalamus in the contralateral hemisphere. Voltage signals were multiplexed and recorded on a USB-6366 board at 400 kHz (NI, Austin, TX, United States). The signals were digitalized at 16 bits by a custom made headstage (Brian Barbarits and Tim Harris, Janelia Farm Research Campus, Ashburn, VA, United States). The signals were demultiplexed into 64-voltage traces, sampled at 25 kHz, and stored by spikeGL (C. Culianu and Anthony Leonardo, Janelia Farm Research Campus, Ashburn, VA, United States). Spikes were sorted offline in JRCLUST (Jun et al., 2017).

The procedure to reconstruct recording locations is the same as before (Wang et al., 2021). To label electrode tracks, the probe was painted with a thin layer of CM-DiI (the chloromethylbenzamido derivative of DiI, dissolved in ethanol; Invitrogen, Waltham, MA, United States). The recording session lasted for about 1 h. Before withdrawing the probe, a small current (20 μA, 1 s, four to six times) was delivered using an electric stimulator (DS3; Digitimer, Welwyn Garden City, United Kingdom) to produce a lesion near the tip of the probe. After recording, the mice were perfused, and their brains were fixed with 4% PFA overnight. Before imaging, the brains were cleared following the uDISCO procedure (Pan et al., 2016). The cleared brains were imaged at a 3 μm × 3 μm × 8 μm spatial resolution (after correction of sample shrinkage). Images from the blue channel (488 nm excitation) were used to segment the CM-DiI signal manually. Images from the red channel (640 nm excitation) were used for registration (see Image processing, visualization, and registration).

### Image Processing, Visualization, and Registration

Customized image processing scripts were written in MATLAB (MathWorks, Natick, MA, United States). The mosaics of each coronal section were stitched based on recorded spatial positions and adjacent overlap by Fiji plugin Grid/collection stitching. Fiji was run within MATLAB *via* MIJ and optimized for parallel processing. Maximum intensity projections and linear contrast adjustments were performed using Fiji. Volume rendering was performed using Imaris Viewer (Bitplane). To remove stripes from acquired images, VSNR10, as a Fiji plugin, was used. After reconstruction, 3D registration was performed to align the images to the mouse brain template in the common coordinate framework (CCFv3; Wang et al., 2020). The images were first down-sampled at a voxel resolution of 10 μm × 10 μm × 10 μm (or 25 μm× 25 μm × 25 μm), to roughly match the size of the template brain. Then, the registration was performed *via* Advanced Normalization Tools (ANTs; Avants et al., 2009). An affine transformation was performed to correct translation, shift, stretch, and rotation. Then, a b-spline transformation was performed to adjust non-rigid inconformity. The deformation field from the red channel was then applied to the blue channel, which allowed us to align the brain together with the probe track and lesion locations to the CCF.

### Visualization and Full Morphology Reconstruction

To trace the full morphology of a single neuron, whole brain data were first transformed from tag image file format (TIFF) to TeraFly format using the Vaa3D-TeryFly program. Morphology reconstruction was then carried out on the TeryFly files within Vaa3D semi-automatically. The final reconstruction is depicted as a single tree structure with multiple branches (no breaks or loops). To visualize the location and spatial distribution of each reconstructed neuron and its dendritic and axonal arbors, the dataset was registered to the CCF (see Image processing, visualization and registration).

Manual annotation of single neurons was performed using Vaa3d (Peng et al., 2010). The multi-resolution whole-brain volume was imported to the Vaa3d TeraFly plug-in, which enabled the visualization of any partial volume centered in a given position of interest at any selected resolution (Peng et al., 2014; Bria et al., 2016). We typically chose several brightest-labeled neurons for annotation. Multiple annotators cooperated to trace the full morphology of single neurons. The first annotator started from the soma and drew each and every process of the neuron by mouse clicking and dragging, using the virtual finger function of Vaa3d, which enabled the accurate reconstruction of neuronal processes (Peng et al., 2014). A second annotator checked the uncertain areas marked by the first annotator and gave correction feedback to the first annotator. Finally, a third annotator, who typically had more experience in the tracing of neuronal morphology, was introduced; if there was any unresolved structure left, the annotator made a final decision. The resulting reconstruction of a single-neuron was saved in the SWC format. Because projection neurons typically have processes with several hundred millimeters of total length and we accurately traced fine axonal collaterals using the virtual finger function of Vaa3D, each neuron, on average, requires about 1 week of full-time work for annotation (the first annotator), with additional time for further checking. For visualization and morphological analysis, the SWC files generated from the annotation based on the 6.3× image volume were first mapped back to the 1.6× image volume through the global coordinate system. Then, the same transformation as the 1.6× image volume was applied to the SWC files to map the coordinates to the CCF. The registered SWC files were used to visualize single-neuron skeletons in the model mouse brain.

### Data and Code Availability

The code for hardware control and imaging generated during this study are available at https://github.com/NeuralCircuits-Behavior/Zhang-Yao-Yin-et-al-2021/^3^. A list of parts for the mLSFM setup is shown in Supplementary Table 1. Data that support the findings are available from the corresponding author upon reasonable request.

## Results

### Multi-Scale Light-Sheet Microscope Setup

**FIGURE 3:**
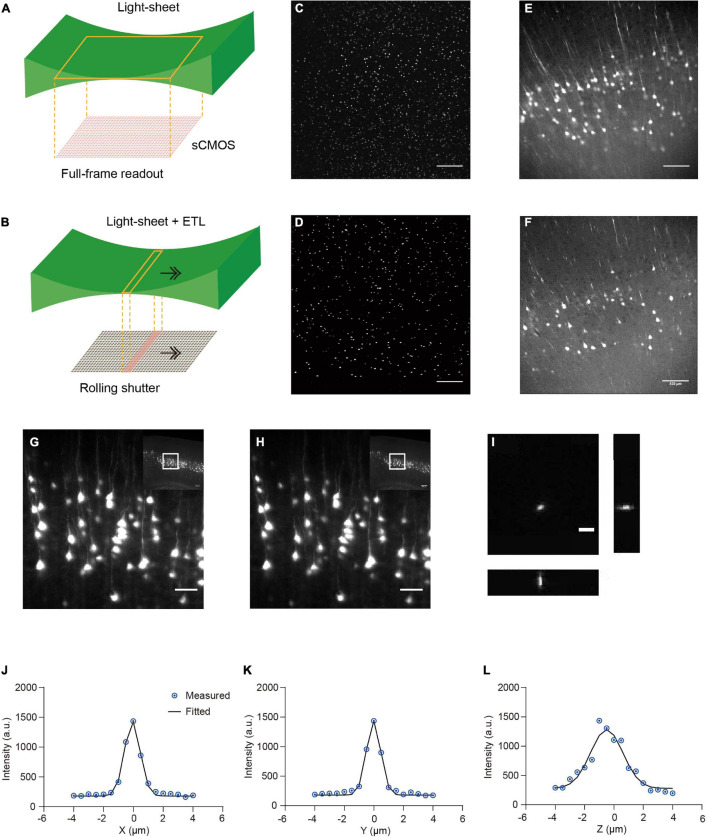
Comparison of stationary light-sheet illumination and tiled light-sheet illumination, non-stop axial scanning during continuous *z*-axis movement, and optical resolution. **(A)** Schematic of the stationary light-sheet and scientific complementary metal–oxide–semiconductor (sCMOS) camera chip. **(B)** Schematic of the axially scanned light-sheet (tiled light-sheet) that moves with slit detection. As the rolling shutter of the sCMOS camera moves synchronously with the light-sheet waist, the final image contains only signal from the waist region, resulting in higher axial resolution across a large field of view (FOV). Arrow, moving direction of light-sheet or rolling shutter. **(C)** Fluorescent beads (1 μm) embedded in 2% agarose gel imaged by the stationary light-sheet. At the top and bottom of the image, beads blur and are dimmer because of being out of focus and increase in sheet thickness. Scale bar in **(C–F)**, 500 μm. **(D)** Fluorescent beads imaged by tiled light-sheet illumination showing small bright dots across the whole FOV. **(E)** An example coronal section of CUBIC-X cleared Thy1-EYFP brain imaged by the stationary light-sheet. **(F)** Same region imaged by tiled light-sheet illumination showing sharper images across the whole FOV. **(G,H)** Fluorescence images of the same region obtained with **(G)** stationary (no moving along Z) imaging and **(H)** non-stop axial scanning. The images are almost identical. Scale bar, 20 μm. **(I)** Fluorescent beads (0.2 μm) embedded in 2% agarose gel were imaged by the multi-scale LSFM. Regions containing single beads were then cropped out and projected along the XY, YZ, and XZ planes. Scale bar, 5 μm. **(J–L)** PSF generated from projected images using the MetroloJ plugin in Fiji. The system has an in-plane resolution of 0.95 μm that is larger than the optical resolution of the system (∼0.6 μm), suggesting that the resolution is limited by the pixel size 0.52 μm. The axial resolution of 2.1 μm is also limited by the axial step size of the motorized stage 1 μm.

We developed a multi-scale light-sheet fluorescence microscopy (mLSFM) system based on selective plane illumination (SPIM; Huisken et al., 2004; Figures 1A, 2). A step motor was attached to a zoom knob to flexibly adjust magnification from 1.26× to 12.6× (Figure 2F). To accompany the changed field of view, a customized shutter was used to automatically adjust the thickness of the light-sheet (Figure 2G). To image a whole mouse brain under near-constant conditions, a vibratome was integrated to section off the imaged top portion of tissue (∼1–4 mm, depending on tissue transparency) when necessary (Narasimhan et al., 2017). To achieve uniform axial resolution across the whole FOV, an electrically tunable lens (ETL) employed in the light-sheet generator was synchronized with the exposure of camera (Figure 2H; Hedde and Gratton, 2016). As the thin portion of light-sheet waist and exposure region of the camera swept through the sample, the images contained signals from the region of narrow light-sheet only, leading to uniform axial resolution across a large FOV (Figures 3A–F). To achieve fast volumetric imaging, a customized linear stage continuously moved along the *Z* axis during acquisition. Compared with non-stop imaging during moving along the *X* axis in the open-top LSFM configuration, movement along the axial direction reduced motion blur, as the axial resolution is typically lower than the in-plane resolution (Narasimhan et al., 2017; Wang et al., 2019). At a speed ranging from 0.1 to 0.16 mm/s with typical exposure time ∼30 ms, there was no evident blur compared with stationary imaging (Figures 3G,H). To characterize the resolution of the system, we imaged 0.2-μm fluorescent beads embedded in agarose gel and found that the system had a resolution of 0.95 μm × 0.95 μm × 2.1 μm along the X-Y-Z directions (Figures 3I–L).

### Whole Brain Imaging From Cellular to Sub-Micron Spatial Resolution

**FIGURE 4:**
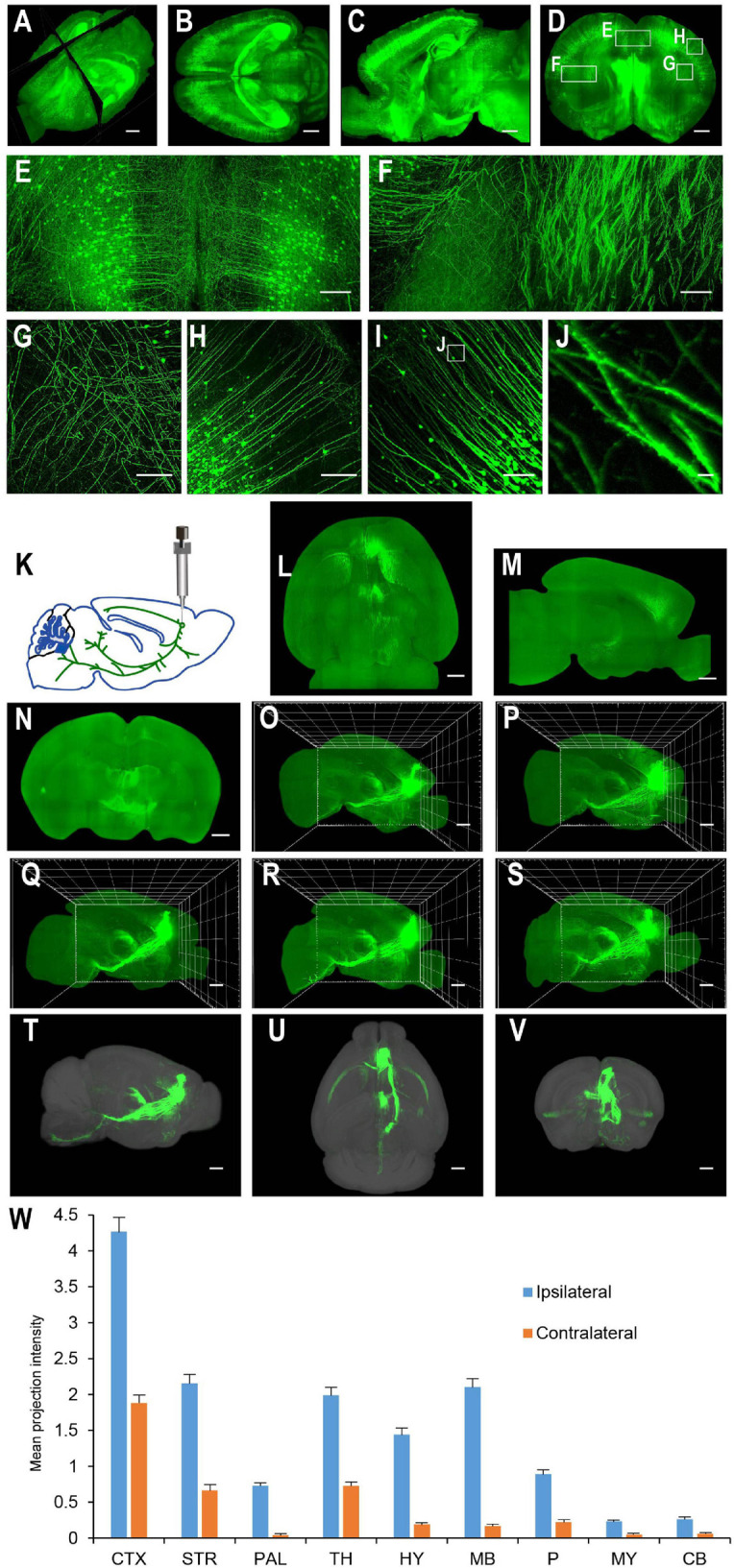
Whole brain imaging of CUBIC-X-cleared Thy1-YFP-H transgenic mouse brains and EGFP virus injected brains. **(A)** Low resolution rendering of a Thy1-YFP-H transgenic mouse brain. The brain was CUBIC-X-cleared. Similar results were observed in four samples. **(B)** Maximum intensity projection (MIP) of a representative horizontal section of the brain in **(A)**. **(C)** MIP of a sagittal section at position indicated in **(A)**. **(D)** MIP of a coronal section at position indicated in **(A)**. **(E–H)** Enlarged view of the boxed regions in **(D)**. **(I)** MIP of a representative coronal section showing apical dendrites of cortical neurons. **(J)** Enlarged view of the boxed region in **(I)** showing dendritic spines. **(K–W)** Whole brain imaging of bulk-injected brain reveals axonal projections of medial prefrontal cortex (mPFC) neurons in different cortical and subcortical areas. **(K)** Anterograde tracing virus (AAV2/1-CAG-tdTomato) is injected in mPFC (AP 1.85, ML 0.4, DV 1.7). **(L–N)** Horizontal, sagittal, and coronal MIPs of a CUBIC-X-cleared brain showing projections of mPFC population neurons. Similar results in *n* = 5 samples. **(O–S)** Sagittal MIPs of five different brains showing similar projection patterns. **(T–V)** Averaged sagittal, horizontal, and coronal MIPs. **(W)** Mean projection intensity approximated by gray value in the ipsilateral and contralateral hemispheres relative to the injection site. CTX, cerebral cortex; STR, striatum; PAL, pallidum; TH, thalamus; HY, hypothalamus; MB, midbrain; P, pons; MY, medulla; CB, cerebellum; M2, secondary motor area; VAL, ventral anterior-lateral complex of the thalamus; VM, ventral media nucleus of the thalamus; MD, mediodorsal nucleus of thalamus; CM, central medial nucleus of the thalamus; PO, posterior complex of the thalamus. Scale bars, 1 mm **(A–D,L–V)**, 200 μm **(E–H)**, 100 μm **(I)**, 5 μm **(J)**.

**FIGURE 5:**
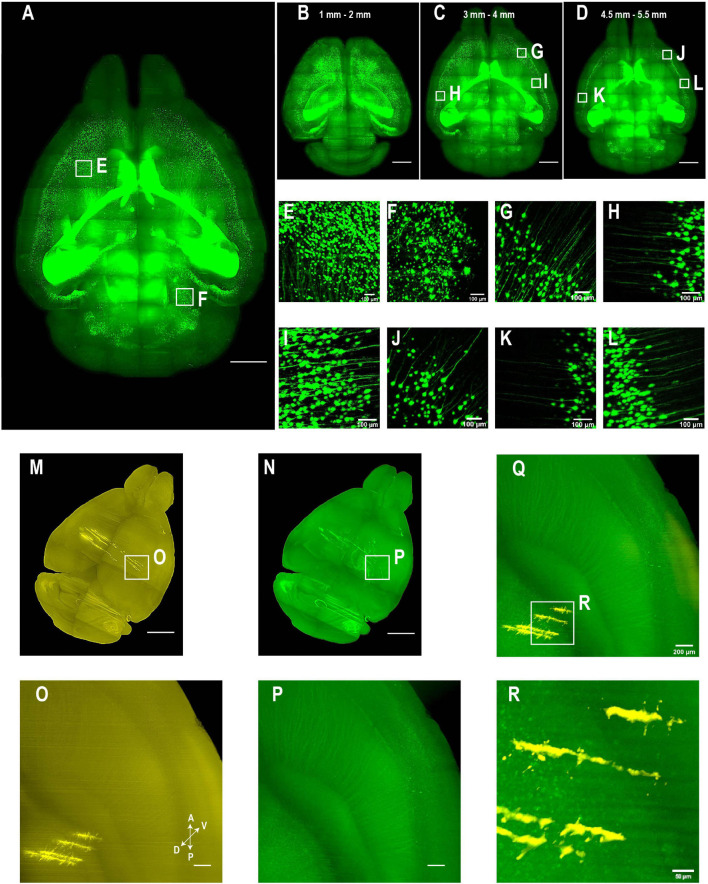
Whole brain imaging of CUBIC-X- and uDISCO-cleared brains without cutting. **(A–L)** Whole brain imaging of a Thy1-EYFP-H transgenic mouse brain cleared by CUBIC followed by MACS RI matching. **(A)** 3D rendering of LSFM images of a cleared Thy1-EYFP-H brain. Similar results in the *n* = 3 samples. **(B–D)** MIPs of horizontal sections at **(B)** 1–2 mm, **(C)** 3–4 mm, or **(D)** 4.5–5.5 mm depth. **(E,F)** Zoomed-in view of boxed regions in **(A)**. **(G–I)** Zoomed-in view of boxed regions in **(C)**. Imaging depth, 3–4 mm. **(J–L)** Zoomed-in view of boxed regions in **(D)**. Imaging depth, 4.5–5.5 mm. **(M–R)** Two-color imaging of uDISCO-cleared brains allows reconstruction of electrode recording locations. **(M)** Whole brain rendering of images acquired by 561-nm excitation (yellow). To label electrode tracks, CM-DiI was painted on the four shanks of a silicon probe prior to insertion. Similar results in the *n* = 2 samples. **(N)** Autofluorescence in the green channel allows good visualization of anatomical features that facilitate whole brain registration to Allen CCF v3. **(O)** Zoomed-in view of the boxed region in **(M)** showing sharp traces of CM-DiI near electrode tips. **(P)** Same region as in **(N)** (green channel). **(Q)** Merged image of **(O,P)** shows electrode localization with anatomical features. **(R)** Zoomed-in view of the boxed region in **(Q)**. Scale bars, 2 mm **(A–D)**, 100 μm **(E–L)**, 1 mm **(M,N)**, 200 μm **(O–Q)**, 50 μm **(R)**.

For applications requiring cellular resolution (i.e., at a few micrometers), imaging by light-sheet fluorescence microscopy with relatively lower magnification can acquire a brighter signal at faster speed with reduced dataset size. We first tested the system for fast whole brain imaging at a cellular axial resolution with intermediate magnification (∼8× effective magnification resulting in micrometer in plane resolution). We imaged CUBIC-X-cleared Thy1-EYFP-H mouse brains at an 0.8 μm × 0.8 μm × 5 μm effective voxel size within 95 min (from start to end of imaging, which included the sectioning time of 30 min; Figures 4A–H; see Supplementary Figure 1 for tissue clearing and Supplementary Table 2 for imaging time analysis; Supplementary Video 1). Imaging at this resolution reveals locations of somata and spatial distribution of axons and dendrites, and thus, is suitable for applications such as tracking bulk projections of axons from a population of neurons (Figures 4K–W). The whole dataset is 550 GB, about 1/20th of that imaged at the resolution for tracing fine axons of individual neurons (Economo et al., 2016). We also imaged mouse brains without sectioning. Under this situation, Thy1-EYFP brains can be imaged at the same voxel size within 60 min (Figures 5A–L).

**FIGURE 6:**
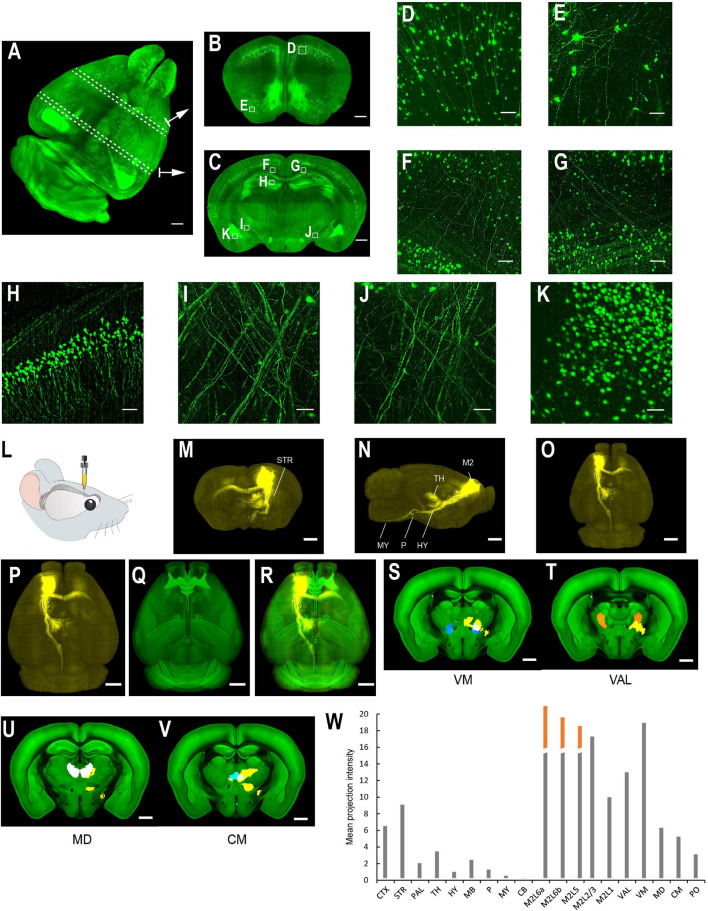
Whole brain imaging of uDISCO-cleared Thy1-YFP-H transgenic mouse brains and EGFP virus injected brains. **(A–K)** Thy1-YFP-H transgenic mouse brains cleared using uDISCO and imaged with the mLSFM setup. **(A)** 3D rendering of images of a uDISCO-cleared brain. Arrows point to example sections. Similar results in the *n* = 5 samples. **(B,C)** MIPs of 1-mm thick coronal sections at positions indicated in **(A)**. **(D,E)** Zoomed-in view of the boxed regions in **(B)**. **(F–K)** Zoomed-in view of the boxed regions in **(C)**. **(L–W)** Whole brain imaging of bulk injected brain (cleared using uDISCO protocol) reveals axonal projections of ALM neurons in different cortical and subcortical areas. **(L)** Schematic of viral injection of AAV2/1-CAG-tdTomato in ALM (AP 2.5, ML 1.5, DV 0.8). **(M–O)** Coronal, sagittal, and horizontal MIPs of a uDISCO-cleared mouse brain with several labeled areas indicated. **(P)** The brain is registered to the Allen common reference brain. **(Q)** Corresponding Allen reference brain. **(R)** Merged image of **(P,Q)**. **(S–V)** Fluorescence signals (yellow) in various thalamic nuclei, including VM, VAL, MD, and CM. Nuclei are color-coded: VM, blue; VAL, orange; MD, white; CM, cyan. **(W)** Mean projection intensity approximated by fluorescence value in brain regions in the ipsilateral hemisphere. Orange bar, contribution from the injection site. CTX, cerebral cortex; STR, striatum; PAL, pallidum; TH, thalamus; HY, hypothalamus; MB, midbrain; P, pons; MY, medulla; CB, cerebellum; M2, secondary motor area; VAL, ventral anterior-lateral complex of the thalamus; VM, ventral media nucleus of the thalamus; MD, mediodorsal nucleus of the thalamus; CM, central medial nucleus of the thalamus; PO, posterior complex of the thalamus. Scale bars, 1 mm **(A–C,M–V)**, 50 μm **(D)**, 100 μm **(E–K)**.

**FIGURE 7:**
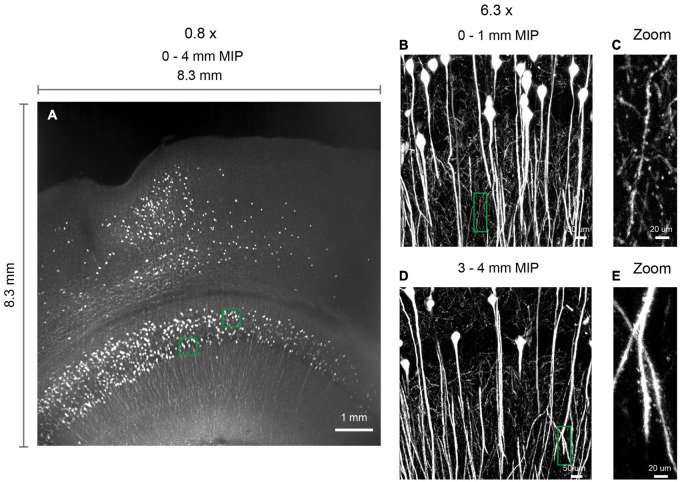
3D imaging of expanded brain slices at submicron resolution. **(A)** 3D rendering of a ∼ 8.3 mm × 8.3 mm × 4 mm volume. Thy1-EYFP-H brain slices were expanded by approximately five times using the MAP protocol. Similar results obtained in two independent experiments. **(B,D)** Zoomed in view of boxed regions in **(A)**. **(C,E)** Zoomed in view of boxed regions in **(B,D)**, demonstrating the ability to visualize spines.

The multi-scale light-sheet fluorescence microscopy is compatible with mouse brains cleared with a variety of clearing methods, such as the aqueous-based, organic-based, and hydrogel-based approaches (such as CUBIC-X, MACS, uDISCO, and MAP; Figures 4–6; Ku et al., 2016; Pan et al., 2016; Murakami et al., 2018; Zhu et al., 2020). We imaged uDISCO-cleared Thy1-EYFP-H mouse brains at an 0.8 μm × 0.8 μm × 5 μm voxel size to visualize the spatial distribution of somata, axons, and dendrites (Figures 6A–K). We also imaged uDISCO-cleared mouse brains with bulk injections in the anterior lateral motor cortex (Figures 6L–W). Consistent with a previous finding (Guo et al., 2017), there are wide-spread projections in thalamic nuclei including VM, VAL, MD, CM, and PO. Tissue expansion methods provide a convenient approach to resolve fine structures with higher spatial resolution (Chen et al., 2015; Ku et al., 2016; Murakami et al., 2018). We also investigated the imaging ability of mLSFM when combined with tissue expansion methods. When a Thy1-EYFP transgenic mouse brain is expanded ∼1.7–2× using CUBIC-X, the resolving power reaches ∼0.5 μm × 0.5 μm × 1 μm, which is comparable with the best available cutting-edge techniques used for brain-wide imaging and tracing of full neuron morphologies (Li et al., 2010; Gong et al., 2013; Economo et al., 2016). Under this condition, small structures, such as spines, are clearly visible (Figures 4I,J). We also cleared and expanded Thy1-EYFP brain slices using MAP (Ku et al., 2016). With expansion by ∼ 5× in each dimension, the resolution reaches 0.2 μm × 0.2 μm × 0.4 μm, providing an easy approach to achieve the high-resolution imaging of neuronal axons and dendritic spines (Figure 7).

### Whole Brain Imaging to Localize Recording Locations

Recording of neural activity is essential in understanding how mammalian brains process information. However, as many brain areas are buried deep from the cortical surface, it is difficult to target specific subcortical areas because of the uncertainty in localization of the recording electrode. To reconstruct marked electrode tracks due to tissue damage or with fluorescent dyes, individual brain slices are typically sectioned, imaged, and then combined to form a 3D brain volume to facilitate the alignment to the model brain in the common coordinate framework (CCFv3; Wang et al., 2020). This process is labor-intensive and prone to slice distortion, and the imaged slices are difficult to be registered perfectly together to form a 3D volume. Thus, whole brain imaging provides a neat way to overcome these problems.

**FIGURE 8:**
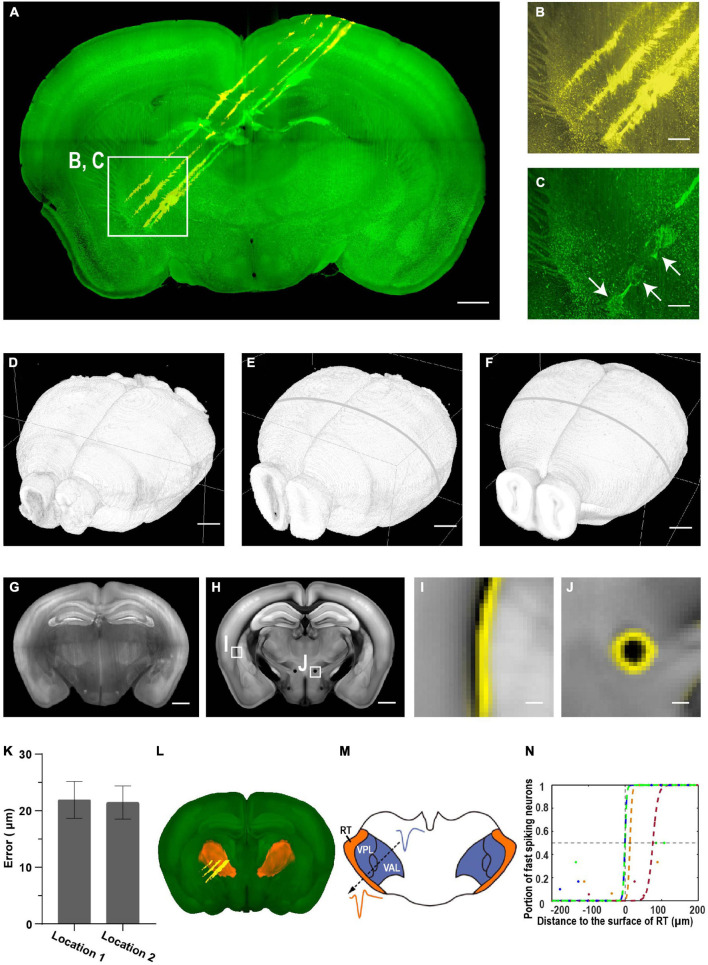
Brain-wide imaging to reconstruct recording locations. **(A)** Schematic of recording from regions near thalamic reticular nucleus (TRN). Two-color imaging was performed to visualize electrode tracks (yellow) and lesion sites (green). Similar results in three of the samples. **(B,C)** Enlarged view of the white rectangular region in **(A)**. Arrows point to three lesion sites. Scale bars, 1 mm **(A)**, 50 μm **(B,C)**. **(D–F)** Surface rendering of images before registration, after registration to the CCF template, and the CCF template. **(G)** Representative coronal slice of the registered brain. Scale bar, 1 mm. **(H)** Corresponding coronal slice of the Allen Reference Brain. Scale bar, 1 mm. **(I,J)** Enlarged view of the boxed regions in **(F)** showing nice match between the registered brain and the reference brain. **(I)** Optic and auditory radiation. **(J)** The mammillothalamic tract. Yellow, boundaries of registered brain. Scale bar, 100 μm. **(K)** Average registration error of boundaries in **(I,J)**. Error bar, SEM (*n* = 3 for each). **(L)** Surface rendering of TRN in the CCF template. **(M)** Example waveforms of neurons from TRN and neighboring thalamic nuclei. **(N)** Portion of narrow spiking neurons increases along the electrode track into TRN. Distance to the boundary at half probability of finding narrow spiking neurons is 22 ± 18 μm (mean ± SEM, *n* = 4). Colored dots, data along different tracks. Colored lines, sigmoid fitting.

To test the accuracy and capacity of mLSFM for electrode localization, we first painted silicon probes with CM-DiI and inserted the probes to target deep brain regions near the thalamus and thalamic reticular nucleus (TRN, Figure 8A). After recording, we delivered a brief electric pulse (20 μA for 1 s, three to four times) to make a small lesion near the tip of the probe (Figures 8B,C; Huo et al., 2020). After fixation, the entire brain was cleared using the uDISCO protocol (Pan et al., 2016). The cleared brain was imaged with dual color at cellular resolution (∼3 μm × 3 μm × 8 μm voxel size after correcting sample shrinkage) within 50 min (including sectioning time of 27 min, Figure 8D) or within 30 min without sectioning (Figures 5M–R; see detailed time analysis in Supplementary Table 3 and Supplementary Video 2). The uDISCO-cleared brains are rigid and have background autofluorescence that facilitates registration to the reference brain in CCF (Figures 8E,F).

We estimated the accuracy of electrode localization using two complementary approaches. First, we compared the boundary difference between the registered brain and reference brain for small internal structures such as fiber tracts (the optic and auditory radiation, the mammillothalamic tract, Figures 8G–J and Supplementary Figure 2). The average distance is 22 ± 1 μm (mean ± SEM, *n* = 6 from three mice, Figure 8K), suggesting that the approach of localizing electrode tracks has a high accuracy. Second, we compared the accuracy of registered electrode tracks with neural physiology landmarks (Figures 8L–N). The TRN is a shell-like structure surrounding other thalamic nuclei, and neurons in the TRN are expressing the neurotransmitter gamma-Aminobutyric acid (GABAergic), while neurons in other thalamic nuclei are mostly glutamatergic. The spike widths formed a bimodal distribution with putative TRN neurons having narrow spike waveforms (Huo et al., 2020). The fraction of narrow spikes increased from near 0 to 1 along electrode tracks from other thalamic nuclei to the TRN. Fitting the transition using a sigmoid function indicates that the approach provides accurate estimation of electrode locations, with a discrepancy of 22 ± 18 μm (mean ± SEM, *n* = 4) between anatomical boundary and electrophysiology landmarks (Figure 8N). Thus, the two approaches provide consistent estimation of recording location, and 3D whole brain imaging has better accuracy than that with imaging individual brain slices (Huo et al., 2020).

### Whole Brain Imaging to Trace Single Projection Neurons in Their Entirety

**FIGURE 9:**
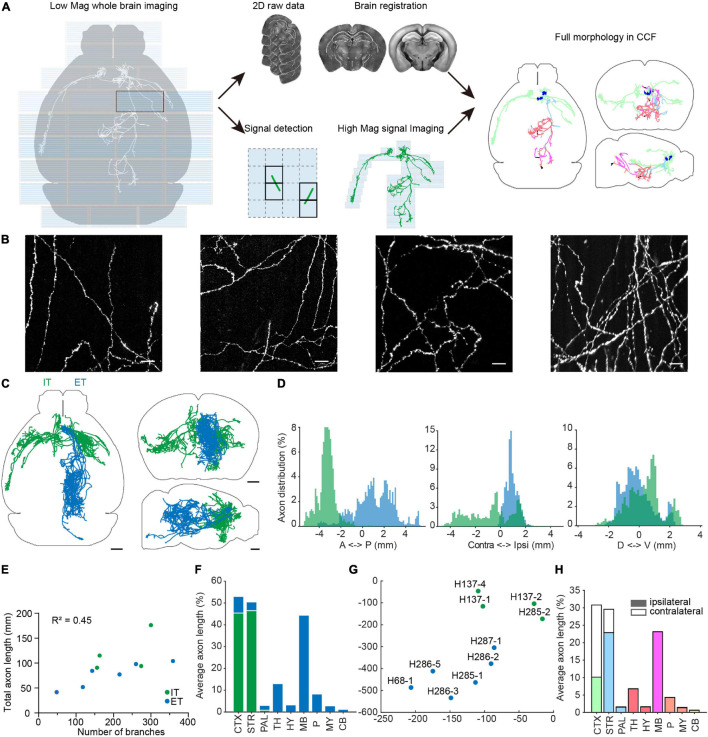
Multi-scale whole brain imaging of sparsely labeled neurons in mPFC. **(A)** Schematic of multi-scale imaging. The process is decomposed into imaging with low magnification for signal detection and imaging signal areas with high magnification. By taking advantage of sparse signals, the efficiency of data acquisition was greatly improved. Finally, the low-magnification whole-brain data were registered to the CCFv3, and the same deformation field was used to register annotations acquired by high magnification. Gray, axonal process before registration. Color, morphology after transformation. Axons located in different brain areas are color-coded. CTX, cerebral cortex; STR, striatum; PAL, pallidum; TH, thalamus; HY, hypothalamus; MB, midbrain; P, pons; MY, medulla; CB, cerebellum. **(B)** Example images of axons from sparsely labeled neurons. **(C)** Overview of traced individual mPFC neurons (*n* = 10). Green, intratelencephalic (IT) neurons; blue, extratelencephalic (ET) neurons. **(D)** Distribution of axons of mPFC IT and ET neurons along the anterior-posterior (AP), medial-lateral (ML), dorsal-ventral (DV) directions. Bregma is chosen as the origin along the AP and ML directions, while the centroid of the brain is chosen as the origin along the DV direction. **(E)** Relationship between total axonal length and number of branches. Same neurons as in **(C)**. **(F)** Brain-wide distribution of axonal processes of mPFC projection neurons. The axonal length in each area is normalized to the total axonal length. The sum of normalized axonal length does not reach 1, as there are *en passant* axons. **(G)** t-Distributed stochastic neighbor embedding (t-SNE) showing clustering of different projection neurons. **(H)** Distribution of axonal processes of reconstructed mPFC neurons in different cortical and subcortical areas. The axonal length in each area is normalized to total axonal length in the indicated areas. Scale bars, 50 μm **(B)**, 1 mm **(C)**.

**FIGURE 10:**
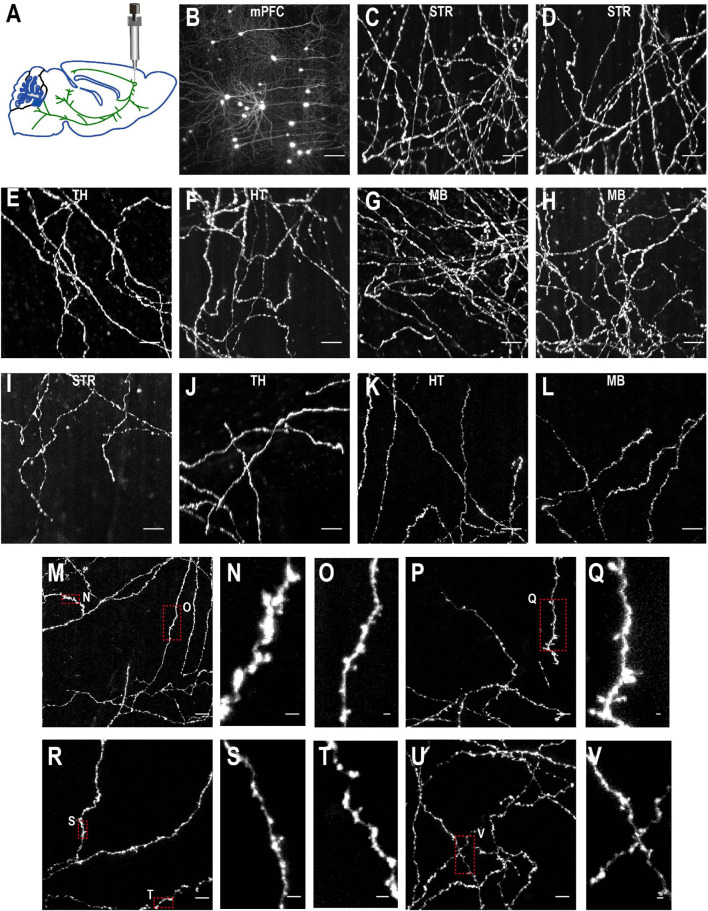
Imaging quality of sparsely labeled mPFC neurons. **(A)** The top-left figure shows schematic of viral injection in mPFC (AP 1.85, ML 0.4, DV 1.7). Same schematic as in Figure 4K. **(B–V)** The rest are MIPs of images in selected regions to show labeled somata and neurites. Enlarged view of axon collaterals in red-dotted regions demonstrates high signal-to-noise ratio of the imaging setup. Scale bars, 100 μm **(B)**, 50 μm **(C–L,M,P,R,U)**, 5 μm **(N,O,Q,S,T,V)**.

Tracing fine axonal projections of single neurons requires imaging the whole brain at high resolution with high contrast (Li et al., 2010; Gong et al., 2013; Economo et al., 2016). The imaging process, even with specialized imaging platforms (Li et al., 2010; Gong et al., 2013; Economo et al., 2016), requires 1 or 2 weeks of continuous imaging that produces 10–20 TBs of data. The long imaging duration poses a great challenge in the mechanical stability of setup as well as in tissue clearing methods (i.e., not to use near saturating concentration of chemicals to avoid precipitation of solutes during extended imaging experiments). In addition, it is difficult to handle a large imaging dataset that together confines the usage of these cutting-edge imaging platforms typically in large neuroscience projects or institutions. As tracing individual axons requires sparse labeling of dozens of neurons (Economo et al., 2016), fluorescent signals only occupy a small portion of the brain. This feature can be combined with mLSFM to enable fast whole brain imaging at high resolution. We first imaged at low magnification by taking advantage of bright signals to efficiently scan for regions with fluorescent signals (see “Materials and Methods”; Figure 9A). The regions with signals are then imaged with high magnification to obtain fine details. Thus, multi-scale imaging can effectively increase imaging speed, and reduce dataset size as well as data acquisition time while maintaining high-resolution high-contrast imaging (Figure 10). We imaged several dozens of sparsely labeled mPFC neurons at 0.3 μm × 0.3 μm × 2.5 μm voxel size within 9 h or 0.3 μm × 0.3 μm × 1 μm voxel size within 13 h (see Supplementary Tables 4, 5 for detailed time analysis). If we have imaged every region of the mouse brain, the imaging time would be approximately three times longer. Thus, with multi-scale imaging, photobleaching is effectively reduced by two-thirds. More importantly, these benefits are not at the cost of imaging quality. Multi-scale LSFM achieves brain-wide high-resolution high-contrast imaging with axon collaterals and axonal terminals having a high signal over noise ratio (Figures 9B, 10).

**FIGURE 11:**
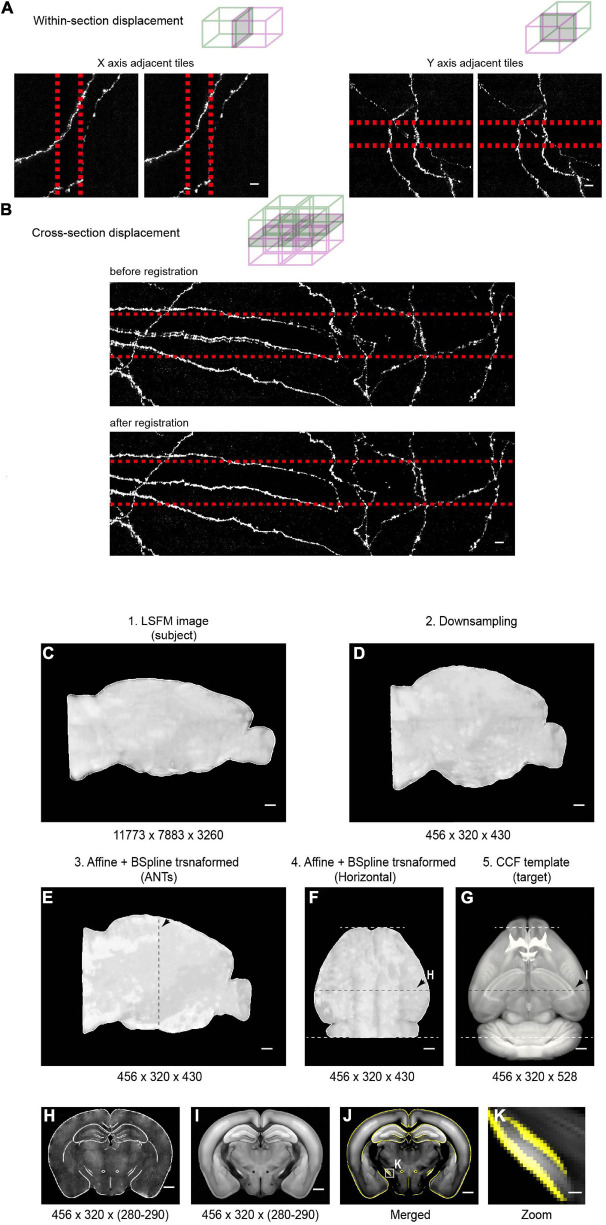
Stitching and registration pipeline. **(A)** In-plane adjacent tiles are stitched by applying a small rigid shift (typically ∼10 pixels between adjacent tiles). Left: MIP of adjacent tiles (along the *X* axis) before (left) and after (right) stitching. Right: MIP of adjacent tiles (along the *Y* axis) before (left) and after (right) stitching. Overlapped regions are indicated by red-dotted lines. **(B)** Stitching along the *Z* axis. Top: MIP of an example region (2 × 2 × 2 tiles) before (top) and after (bottom) non-rigid stitching. Before stitching, there were clear mismatches that were corrected after stitching. **(C–E)** Pipeline of registration of whole-brain imaging data to the Common Coordinate Framework (CCF v3) for CUBIC-X-cleared brains. The whole brain 3D volume **(C)** is down-sampled to 25 μm × 25 μm × 25 μm voxels **(D)**, the same voxel size as the Allen Reference Brain. Then affine transformation is applied in the software ANTs to register the down-sampled brain to the Allen Reference Brain **(E)**. Arrows in **E–G** indicate the position of the example coronal section shown in **H–I**. **(F,G)** Horizontal view of the registered brain and the corresponding reference brain. **(H,I)** Coronal slice at the position indicated by arrows in **(F,G)**. **(J)** Overlay of the boundaries of registered brain (yellow) on the Allen Reference Brain. **(K)** Enlarged view of the boxed region in **(J)** shows nice match between the registered brain and the reference brain. Scale bars, 5 μm **(A,B)**, 1 mm **(C–J)**, 100 μm **(K)**.

**FIGURE 12:**
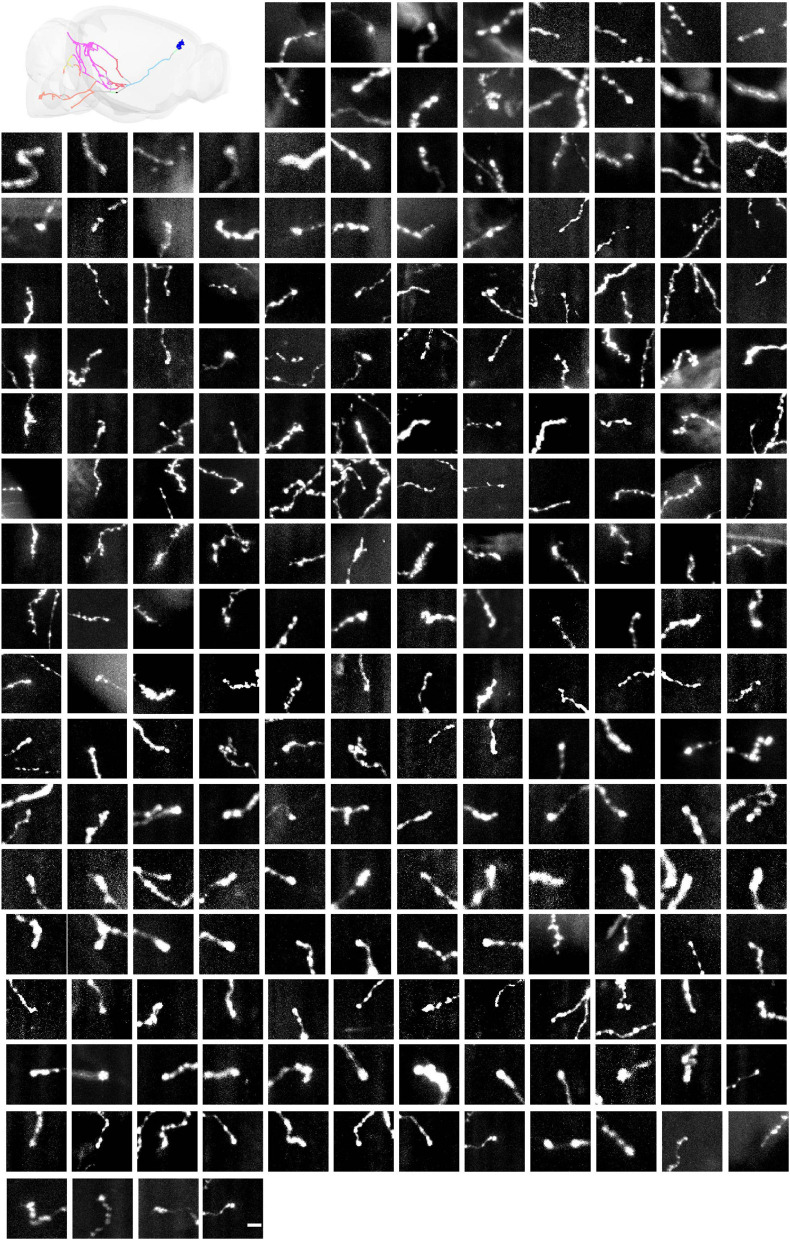
All axonal terminals of one mPFC neuron. Maximum intensity projection (raw coronal images) of all the axonal terminals (*n* = 212) belonging to the mPFC neuron. Axons in different brain areas are color coded (same scheme as in Figure 9A). Scale bar, 5 μm.

**FIGURE 13:**
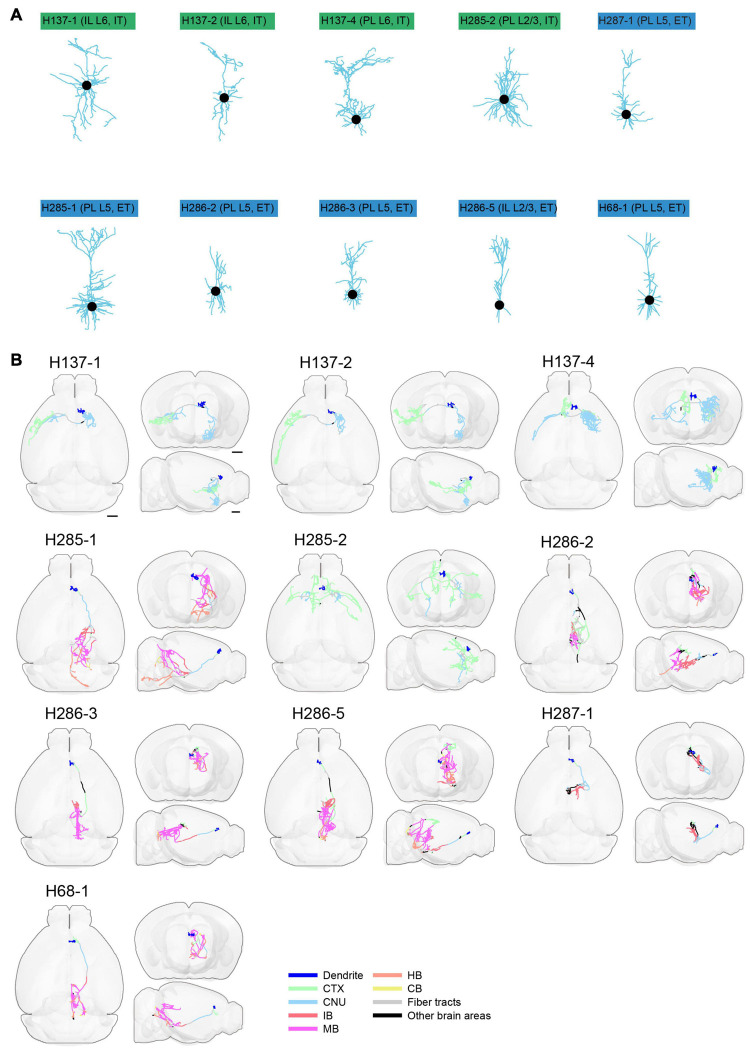
Morphology of reconstructed mPFC neurons. **(A)** Morphology of dendrites of different types of neurons in mPFC (*n* = 10). Dendritic morphology of IT (green) and ET (blue) neurons are shown. **(B)** Horizontal, coronal, and sagittal views are shown for each neuron in **(A)**. Dendrites are shown in blue. Axons in each indicated brain areas are color-coded using the same scheme as in CCFv3. CTX, cerebral cortex; CNU, cerebral nuclei; IB, interbrain; MB, midbrain; HB, hindbrain; CB, cerebellum. Scale bar, 1 mm.

**FIGURE 14:**
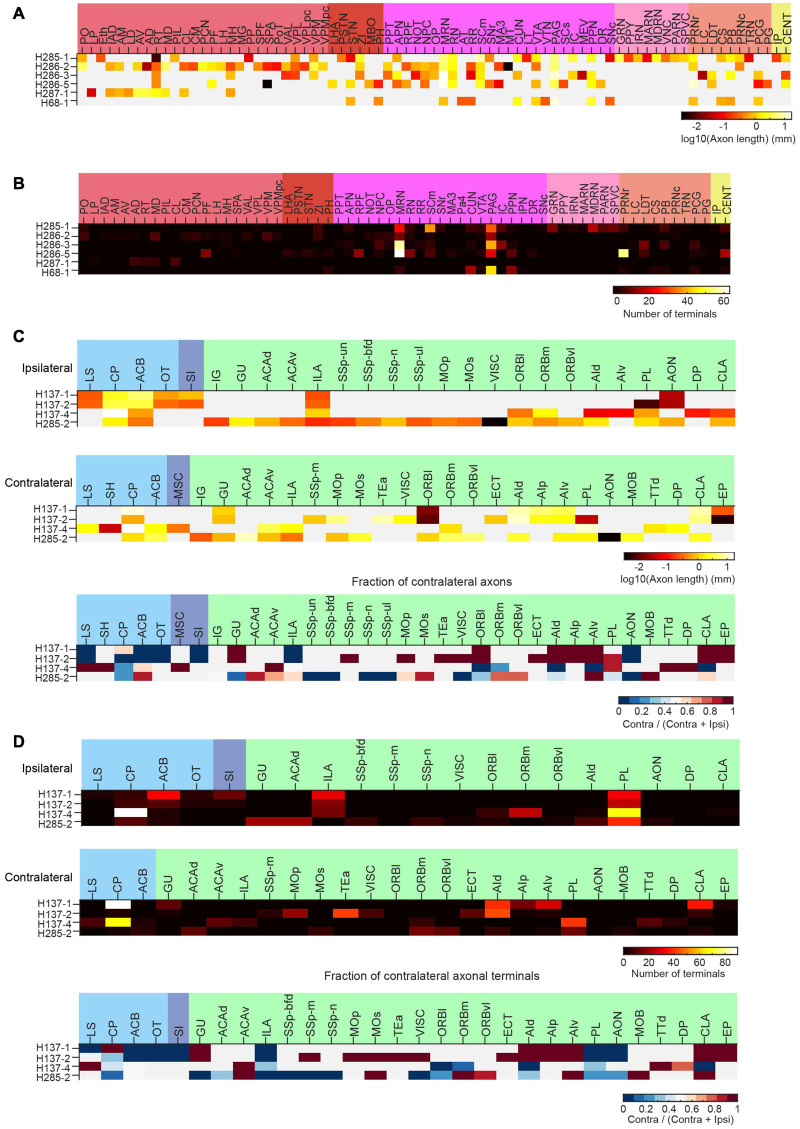
Long-range projections of mPFC ET and IT neurons. **(A)** Axonal length of individual ET neurons in different cortical and subcortical areas in the ipsilateral hemisphere. **(B)** Number of axonal terminals of individual ET neurons in the ipsilateral hemisphere. There are no terminals in the contralateral hemisphere for the reconstructed ET neurons. **(C)** Axonal length of individual IT neurons in different cortical and striatal areas in the ipsilateral (top) or contralateral (middle) hemisphere. The normalized fraction of axonal length in the contralateral hemisphere (to total axonal length) is shown in the bottom row. **(D)** Number of axonal terminals of individual IT neurons in the ipsilateral (top) or contralateral (middle) hemisphere. The normalized fraction of axonal terminals in the contralateral hemisphere (to total axonal length) is shown in the bottom row. There are more terminals in the ipsilateral hemisphere.

To facilitate whole-brain tracing of single neurons, individual tiles were stitched together using their overlapped regions (5% in x-y and 10% along z) (Figures 11A,B), and then the assembled brain was registered to the common coordinate framework (Figures 11C–K). At the resolutions we used, we can successfully trace individual neurons with their morphology in entirety, as every axonal terminal is clearly visible (212 total termini for the example neuron in Figure 12). We traced the full morphology of 10 mPFC projection neurons (Supplementary Video 3). The dendritic and axonal processes of these neurons span several millimeters along the AP, DV, and ML directions (Figures 9C,D, 13, 14). The average axonal length is 95 ± 33 mm (mean ± SEM, *n* = 10), with some neurons reaching nearly 200 mm (Figure 9E). The total axonal length is linearly correlated with the number of branches (Figure 9E). At the individual neuron level, there are, on average, 204 ± 92 (mean ± SEM, *n* = 10) branches, hierarchically organized in a dendrogram with varied depths (Supplementary Figure 3). There are several types of cortical projection neurons classified based on their distinct projection targets (Harris and Shepherd, 2015). Consistently, the intratelencephalic (IT) neurons mainly project within the cortex and striatum, while extratelencephalic (ET) neurons project to a set of nuclei in the midbrain and hindbrain, including the thalamus, hypothalamus, midbrain, pons, and medulla (Figures 9F, 14). Indeed, the t-distributed stochastic neighbor embedding (t-SNE) distribution shows that IT and ET neurons form distinct clusters (Figure 9G). Both IT and ET neurons have more terminals in the ipsilateral hemisphere (Supplementary Figure 3). The widespread projection pattern based on single neurons qualitatively recapitulates the pattern based on bulk labeling of mPFC neurons (imaged using the same setup at slightly reduced resolution by taking advantage of multi-scale imaging, Figure 9H and Supplementary Figure 4).

**FIGURE 15:**
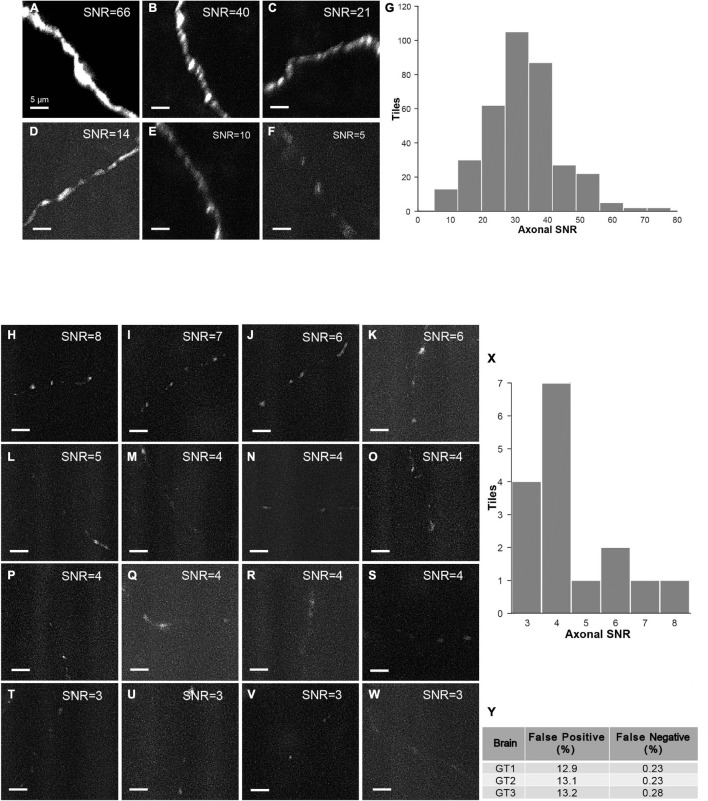
Characterization of false-positive and false-negative rates. **(A–F)** Axons in randomly selected tiles (maximum intensity projected) from three ground-truth brains. **(G)** Histogram of SNR of randomly selected tiles with signals (*n* = 355). **(H–W)** Axons in false-negative tiles. Signals in these tiles have very low SNR. **(X)** Histogram of SNR of false-negative tiles from three ground-truth brains (total tile number = 16). **(Y)** Characterization of false positive rate and false negative rate in three ground-truth brains (GT1, GT2, and GT3). Scale bars, 5 μm **(A–F,H–W)**.

The strategy of mLSFM depends on the successful detection of signals at low resolution that is facilitated by the unique advantage of multi-scale imaging in light-sheet microscopy. To quantify the accuracy of signal detection, we further imaged all regions in a set of brains after multi-scale imaging (Figure 15). False positive rate, defined as the fraction of imaged tiles without fluorescent signals, reached 13.1% on average. False negative rate was limited to 0.25%, equivalent to 5 out of 2,000 tiles in the imaged brain samples. These undetected tiles have a low signal-to-noise ratio (SNR) of 4.5 ± 1.5 (mean ± SD, *n* = 16 in three brains), as these regions typically have faint axons from weakly labeled neurons (the viral delivery strategy does not label neurons with uniform brightness, Figures 15H–Y). For comparison, signals in other regions have an SNR of 34.5 ± 11.6 (mean ± SD, randomly selected 355 tiles from three brains). Low fluorescence signals render the tracing of full neuronal morphologies almost impossible; thus, the undetected signals do not affect the pipeline of brain-wide imaging and reconstruction. We also verified manually that traced neurons have a nearly complete morphology with every terminal to be clearly visible.

## Discussion

Whole-brain imaging provides crucial information, such as somata distribution, dendritic morphology, projection patterns, and neural activity patterns. Deciphering the structure and function of neural circuits requires whole brain imaging at multiple scales (Huisken et al., 2004; Ahrens et al., 2013; Keller and Ahrens, 2015; Power and Huisken, 2017; Harris et al., 2019; Winnubst et al., 2019; Ueda et al., 2020a). Imaging of activated neurons using immediate early gene markers or genetically encoded calcium indicators at cellular resolution (∼5 μm) provides clues of functionally defined circuits across mammalian brains (Ahrens et al., 2013; Renier et al., 2014). Systematic mapping of axonal projections from defined brain areas and cell types at micrometer resolution reveals organization principles of the cortex, thalamus, and their interconnections (Harris et al., 2019). Brain-wide imaging of sparsely labeled neurons at synaptic resolution demonstrates intricate arborization of individual neurons across brain areas (Winnubst et al., 2019; Peng et al., 2020). As different experiments require imaging at different resolutions, imaging at the right spatial scale not only reduces imaging time and dataset size but also reduces difficulty in data processing. We provide theory and verification to demonstrate that compared with wide-field imaging, light-sheet microscopy is especially suitable for multi-scale imaging (Figure 1). With real-time multi-scale imaging, we have imaged cleared whole mouse brains from cellular to synaptic resolutions all in a few hours to fulfill different experimental needs.

Tissue-clearing methods use mechanisms of lipid removal and tissue homogenization to enable biological tissue transparency (Erturk et al., 2012; Chung et al., 2013; Susaki et al., 2014; Hama et al., 2015; Richardson and Lichtman, 2015; Pan et al., 2016; Murakami et al., 2018; Ueda et al., 2020b; Zhu et al., 2020). The mLSFM is compatible with all major tissue-clearing techniques such as aqueous-based, organic-based, and hydrogel-based approaches (Figures 4–7). High-resolution imaging requires the thickness of excitation light to be very thin and consequently limits FOV due to the inverse relationship between FOV and light-sheet thickness (Gao, 2015; Voigt et al., 2019). Thus, light-sheet thickness and FOV are compromised in conventional LSFM to enable efficient imaging. Therefore, a wide range of techniques with optimized light-sheet generation such as using Airy beam light-sheet and lattice light-sheet, has been developed (Saghafi et al., 2010; Chen et al., 2014; Vettenburg et al., 2014; Chang et al., 2019). Tiling light-sheet methods use a spatial light modulator, electrical tunable lens, TAG lens, or remote focusing voice coil to tile a relatively small but thin light-sheet to illuminate whole samples, enabling high spatial resolution and good optical sectioning capability within an FOV larger than the light-sheet itself (Schacht et al., 2010; Baumgart and Kubitscheck, 2012; Dean and Fiolka, 2014; Dean et al., 2015; Gao, 2015; Hedde and Gratton, 2016; Voigt et al., 2019; Chen et al., 2020). In mLSFM, we used an electrical tunable lens to synchronize the sweeping light-sheet waist with the exposure rolling-shutter of sCMOS sensors to achieve a more uniform axial resolution. The concept of multi-scale imaging is focused on the detection optical path, and thus, is inherently compatible with different configurations of illumination, such as digitally scanned light sheet generation and various axially sweeping approaches (Keller et al., 2008; Dean et al., 2015; Hedde and Gratton, 2016; Voigt et al., 2019; Chen et al., 2020), to allow for seamless integration. To have a constant quality of imaging, we have integrated a vibratome to section off the imaged top sections (Figure 2). Sectioning is necessary to achieve a constant image quality throughout the *Z*-axis, which can be very challenging to have with intact brains. Sectioning can induce small deformation of tissue (at a micrometer scale). However, the large overlap between sections (100–300 μm as the imaging depth can reach 2–3 mm) provides enough information for stitching. Sectioning also breaks one intact brain sample into multiple slices. However, as each slice is at least a few hundred micrometers thick, brain sections can be collected for future immunostaining or imaging.

By achieving high-speed imaging of cleared mouse brains from cellular to synaptic resolutions to precisely locate sites of extra-cellular electrodes, or to map long-distance axonal projections across multiple brain areas, or to reconstruct complete axonal morphology, the versatile mLSFM proves to be a capable, reliable, and flexible imaging system. The mLSFM approach can image complete mouse brains at 0.8 μm × 0.8 μm × 5 μm resolution within 95 min (or 60 min without tissue sectioning), faster than reported whole brain imaging methods at similar resolutions [8 h (Narasimhan et al., 2017) and 2.4 h (Seiriki et al., 2017)]. The mLSFM system can also image brain-wide signals at 0.3 μm × 0.3 μm × 1 μm resolution within 13 h, an order of magnitude faster than MOST and serial two-photon tomography (Li et al., 2010; Gong et al., 2013; Economo et al., 2016). This further demonstrates that real-time data analysis, combined with instrument control, can achieve previously infeasible flexibility and augment the performance of specific imaging systems (Peng et al., 2014; Long et al., 2017; Chen et al., 2021). Efficient acquisition of whole brain dataset to track fine axonal arbors to their termini allows tracing of single-neuron morphology, facilitating rapid characterization of cell types (Harris and Shepherd, 2015; Harris et al., 2019; Winnubst et al., 2019; Peng et al., 2020).

## Conclusion

We provided theory and experiments to demonstrate that light-sheet fluorescence microscopy is especially suitable for multi-scale imaging. The multi-scale light-sheet microscopy developed here combines tiling of light-sheet, automatic zooming, periodic sectioning, and tissue expansion, and achieves a constant quality of brain-wide imaging from cellular to sub-micron spatial resolution rapidly in a few hours. The approach and system will facilitate whole-brain imaging of structures to gain further insights into the complexity of brain anatomy.

## Data Availability Statement

The original contributions presented in the study are included in the article/Supplementary Material, further inquiries can be directed to the corresponding author.

## Ethics Statement

The animal study was reviewed and approved by the Institutional Animal Care and Use Committee (IACUC) of Tsinghua University.

## Author Contributions

ZZ built the imaging setup, acquired whole brain imaging data, and traced single neurons. ZZ and XXY wrote the control program. TH, RJ, and ZZ performed the virus labeling and mouse brain clearing. YH and XXY performed the electrophysiology experiments and analysis. TH, ZD, and HP set up the tracing pipeline using Vaa3d. XY, XXY, and ZZ performed the data analysis. ZG conceived and supervised the project. ZZ and ZG wrote the article, with comments from other authors. All authors contributed to the article and approved the submitted version.

## Conflict of Interest

The authors declare that the research was conducted in the absence of any commercial or financial relationships that could be construed as a potential conflict of interest.

## Publisher’s Note

All claims expressed in this article are solely those of the authors and do not necessarily represent those of their affiliated organizations, or those of the publisher, the editors and the reviewers. Any product that may be evaluated in this article, or claim that may be made by its manufacturer, is not guaranteed or endorsed by the publisher.

## Footnotes

1
http://n2t.net/addgene:105553

2
http://n2t.net/addgene:51502

3
https://doi.org/10.5281/zenodo.5154882
