# Supplementary material for: Multi-Scale Light-Sheet Fluorescence Microscopy for Fast Whole Brain Imaging
Source: Front Neuroanat. 2021 Sep 24;15:732464. doi: 10.3389/fnana.2021.732464 (PMC8497830; doi:10.3389/fnana.2021.732464)
Supplement: Supplementary file 4 [file Data_Sheet_1.docx]

Supplementary Material

# Supplementary Figures and Tables

## Supplementary Figures


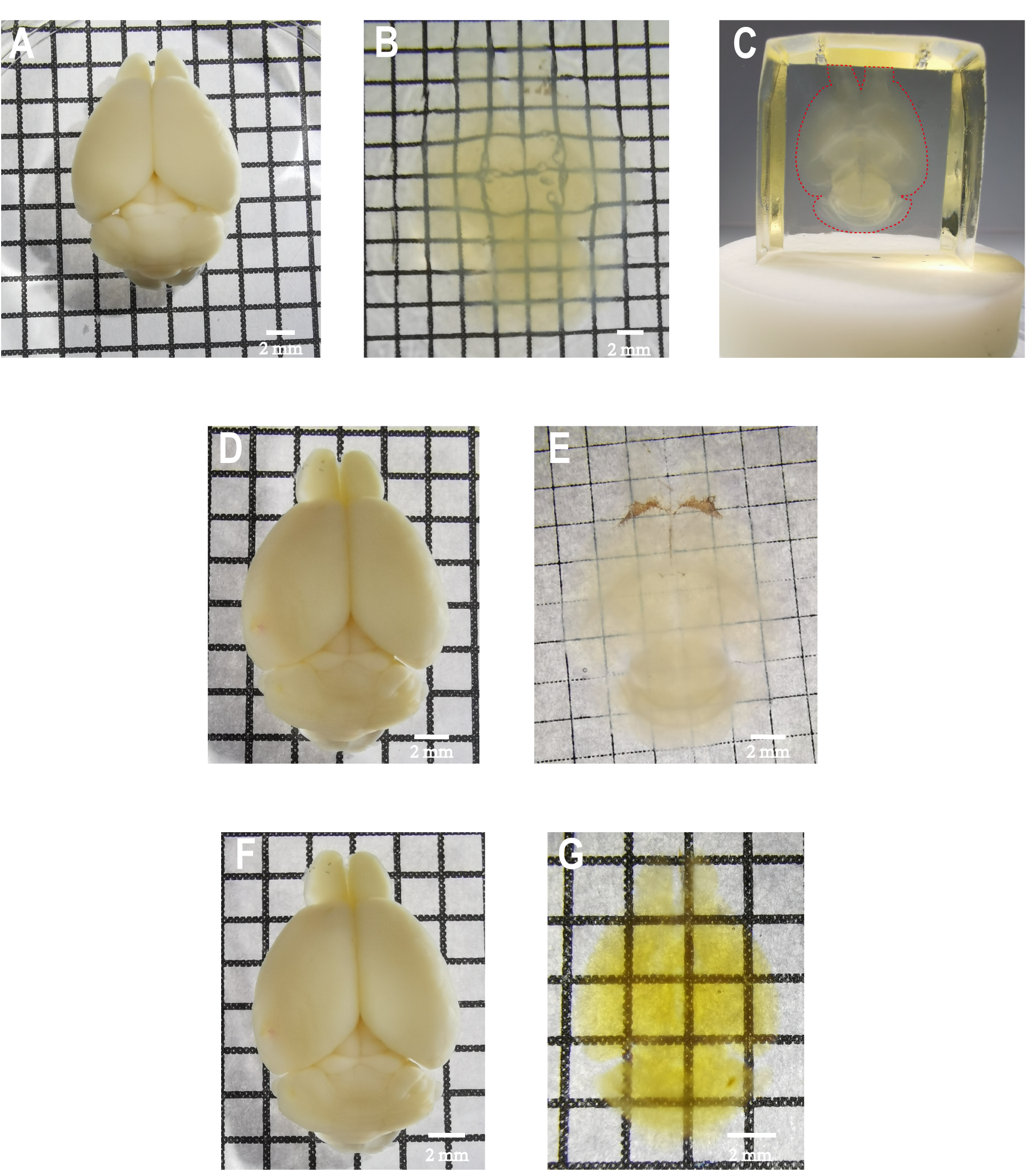


**Supplementary Figure 1.** **Sample preparation of cleared brains using various clearing protocols. (A)** A mouse brain after fixation in 4% PFA. **(B)** The same brain in (A)after clearing by CUBIC-X protocol. **(C)** The CUBIC-X cleared brain embedded in 4% agarose gel dissolved in CUBIC-X2 solution. **(D-E)** A mouse brain before and after clearing by MACS protocol. **(F-G)** A mouse brain before and after clearing by uDISCO protocol.


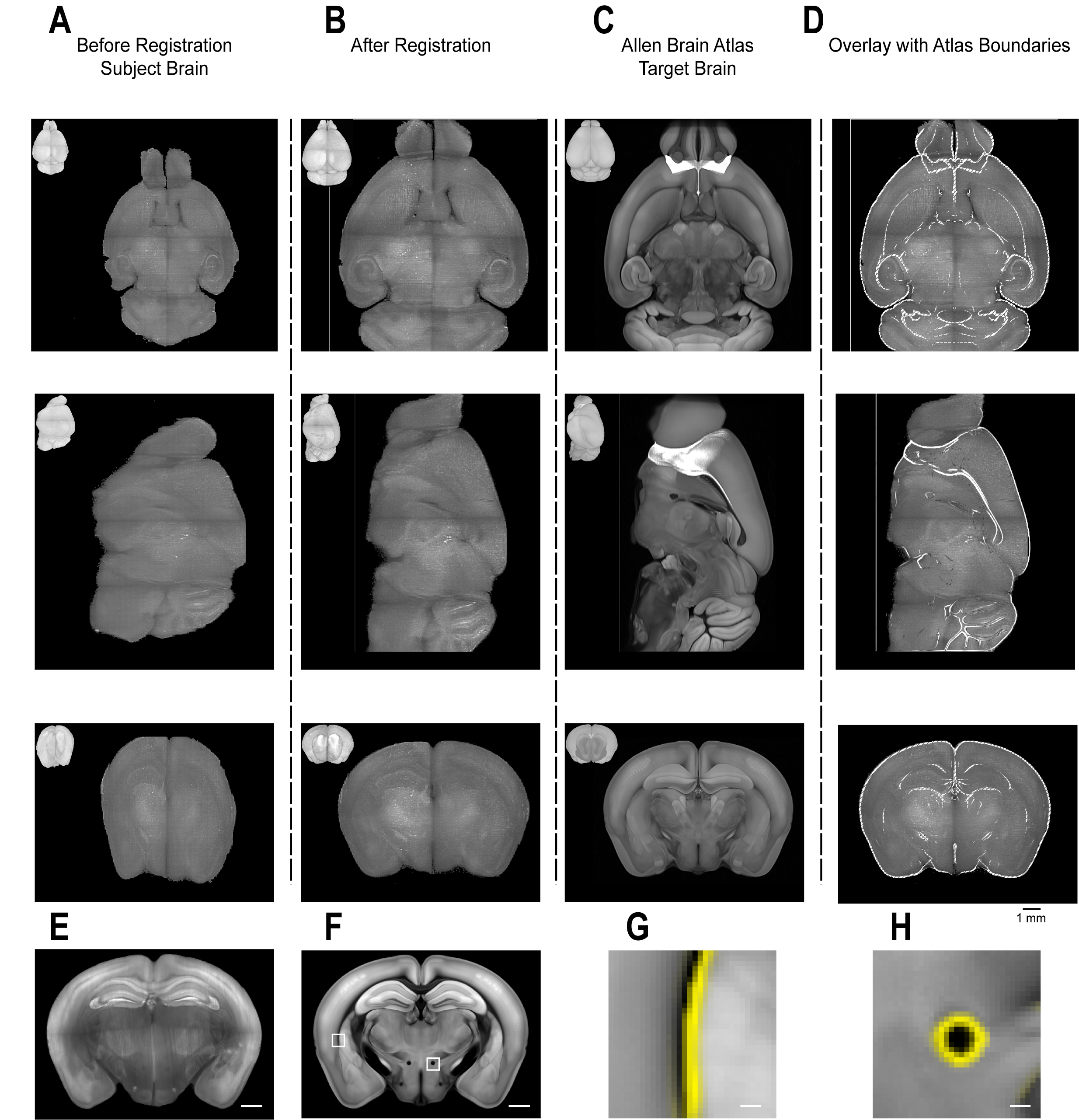


**Supplementary Figure 2 | Registration of whole-brain imaging data to the Common Coordinate Framework (CCF v3) for uDISCO cleared brains. (A)** The horizontal, sagittal and coronal MIPS of a uDISCO cleared brain before registration. **(B)** The horizontal, sagittal and coronal MIPS of the same brain in **A** after registration to CCF v3. **(C)** The corresponding horizontal, sagittal and coronal MIPS of the Allen Reference Brain. **(D)** The overlays of the registered brain and the boundaries of Allen Reference Brain (white curves) showing high accuracy of registration. **(E)** A representative coronal slice of the registered brain. Same as Figure 8G. Scale bar, 1 mm. **(F)** The corresponding coronal slice of the Allen Reference Brain. Same as Figure 8H. Scale bar, 1 mm. **(G-H)** The enlarged view of the boxed regions in **F** showing nice match between the registered brain and the reference brain. Yellow, boundaries of registered brain. Same as Figure 8I, J. Scale bar, 100 μm.


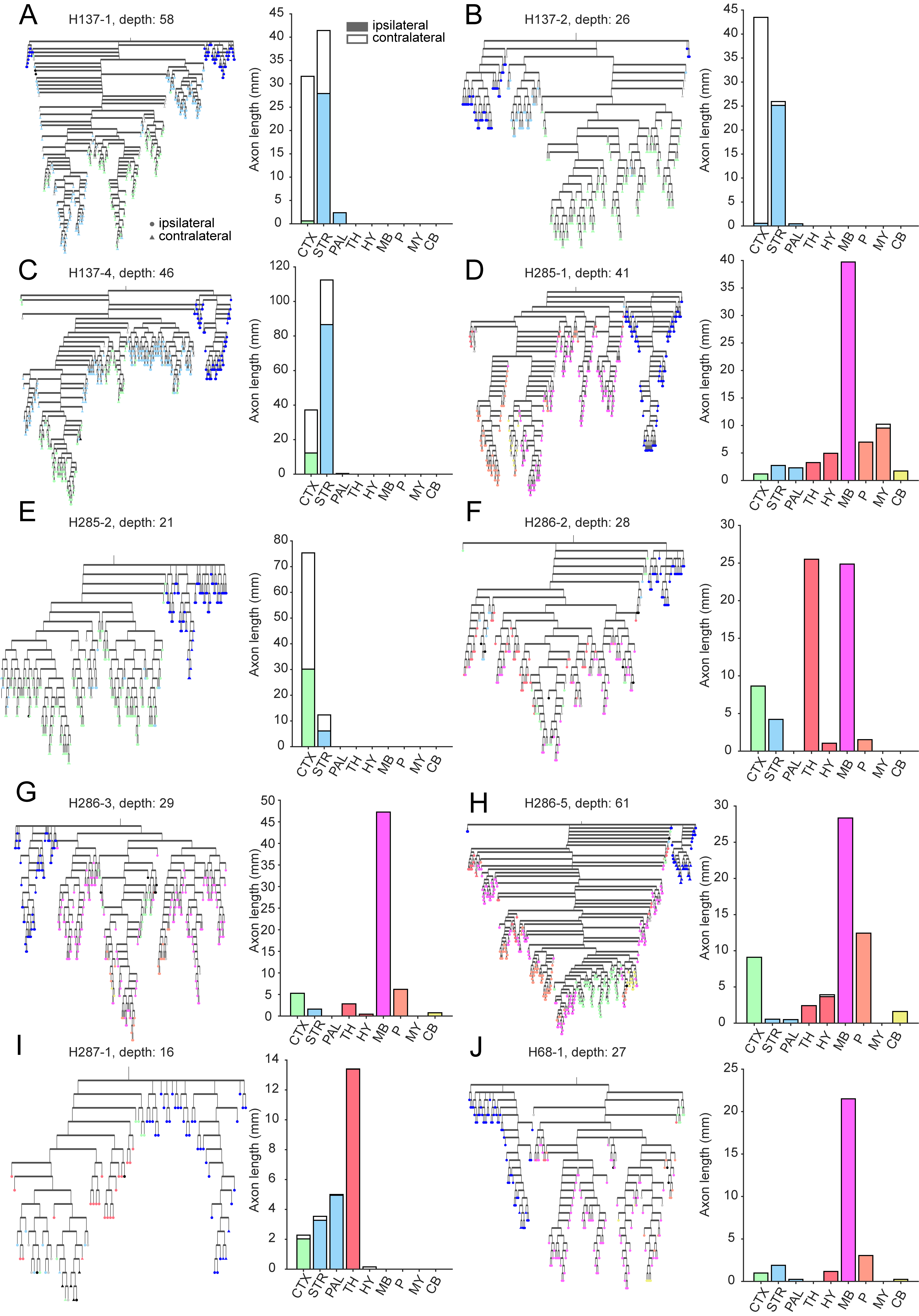


**Supplementary Figure 3. Topology of reconstructed mPFC neurons. (A-J)** Left, dendrogram of individual neurons. Length of axons and dendrites are not proportional in the plotted dendrogram. Depth represents the maximum number of branches along one axonal path to terminal. Triangles, terminals of contralateral axons. Circles, terminals of ipsilateral axons. Right, axonal length in each indicated brain area. Color scheme is the same as in the left. CTX, Cerebral cortex; STR, Striatum; PAL, Pallidum; TH, Thalamus; HY, Hypothalamus; MB, Midbrain; P, Pons; MY, Medulla; CB, Cerebellum.

**Supplementary Figure 4. Combination of single neuron morphologies qualitatively recapitulates population-level neuronal projection patterns. (A)** Brain-wide projection of traced individual mPFC neurons (n = 10). Green, IT neurons; Blue, ET neurons. **(B)** Distribution of axons of mPFC IT and ET neurons along the anterior-posterior (AP), medial-lateral (ML), dorsal-ventral (DV) directions. **(C)** The horizontal, sagittal and coronal MIPs of a CUBIC-X cleared brain with bulk injection in mPFC (averaged over 5 samples). **(D)** The distribution of axonal projections of bulk labeled mPFC neurons in different cortical and subcortical regions. The projection strength is approximated by the number of voxels detected in each area and then normalized to the total number of voxels in indicated areas.

## Supplementary Tables

| Item # | Part name | Product # | Quantity | Vendor |
| --- | --- | --- | --- | --- |
| 1 | Vibratome | VT1200 | 1 | Leica |
| 2 | Laser system 488 & 561 & 640 nm | OBIS 488 & 561 & 640 nm-100 mW | 1 | Coherent |
| 3 | Acousto-optic tunable filter | AOTFnC-400.650-TN  MDS8C-B66-22-74.158 | 2  1 | AA Opto-Electronic |
| 4 | Light-sheet generator stage (x 2) | MTS204  SC102 | 2 (per side)  1 (per side) | Beiguangshiji, Beijing |
| 5 | Illumination path (Light-sheet generator x 2)  Collimator  Beam expander-1  Beam expander-2  Cylindrical lens-1  Cylindrical lens-2  Electrically focus-tunable lens  Drivers/power supply of ETL  Cemented achromatic doublets  Cage system adapters  30mm cage right-angle kinematic mirror mount with tapped cage rod holes  Broadband dielectric mirror  Objective  Objective holder | CFC-11X-A (optic mount: CXY1)  ACN254-040-A (f = -40.0 mm, optic mount: CP06/M),  AC254-200-A (f = 200.0 mm, optic mount: CP02T/M)  LJ1810L2 (f = 25.00 mm, H = 10.00 mm, L = 20.0 mm, optic mount: CYCP/M)  LJ1703RM-A（f = 75 mm, optic mount: CP06/M)  EL-10-30-VIS-LD (Focus range: +50 mm to +120 mm, Current 0-300 mA, optic mount: CP12)  LD1255R [250 mA,3.3 V, optical table mounting plate: LD1255P, Power supply: PS-12DC-EU (± 12 VDC,220-240 VAC) Cable: LD1255-CAB]  AC508-200-A (f = 200.0 mm, optic mount: LCP01/M)  LCP02/M  KCB1    BB2-E02 (coating = 400-750 nm)  MVPLAPO 1X (WD = 65 mm, NA= 0.25, FOV: 34.9-3.5 mm)  Inner diameter: 60.1mm, 64.5mm | 1 (per side)  1 (per side)  1 (per side)  1 (per side)  1 (per side)  1 (per side)  1 (per side)  1 (per side)  1 (per side)  1 (per side)  1 (per side)  1 (per side)  1 (per side) | Thorlabs  Thorlabs  Thorlabs  Thorlabs  Thorlabs  Optotune (Edmund optics)  Thorlabs  Thorlabs  Thorlabs  Thorlabs  Thorlabs  Olympus  Customized |
| 6 | Detection path (MVX-ZB10)  Zoom body  Tube lens  C-mount adapter  Revolving nosepiece  Motorized focus unit  Control unit  Hand switch  Power supply ac adapter  Objective lens  Stepping Motor  Dipping cap | MVX-ZB10  MVX-TLU  MVX-TV1XC  MVX-2RE  SZX2-FOA  SZX2-MDCU  SZX2-MDHSW  U-ACAD4515-1-5  MVPLAPO 2XC  (WD = 20 mm, NA = 0.5, FOV: 17.6-1.7 mm)  17HS0401-18B (42mm Stepping Motor)  Customized | 1  1  1  1  1  1  1  1  1  1  1 | Olympus  Olympus  Olympus  Olympus  Olympus  Olympus  Olympus  Olympus  Olympus  RDG  Customized |
| 7 | Filter wheel | W32  Lambda 10-B | 1  1 | Sutter Instrument |
| 8 | Filter | 59022m EGFP/mCherry  ET655lp  ZET405/488/561/640m | 1  1  1 | Chroma  Chroma  Chroma |
| 9 | sCMOS camera | Orca-Flash 4.0 V2 | 1 | Hamatsu |
| 10 | Optical table | QF12-09 (1200 x 900 x 100 mm) | 1 | Liansheng |
| 11 | 3D motorized stage | HGTA0650A  HGTA06100A | 2  1 | Henggong,  Beijing |
| 12 | Sample holding chamber | Chamber Base  Out distance: 180mm*95mm*45mm  Inner distance: 176mm*91mm*35mm  Wall thickness: 2mm  Base thickness: 10mm  Chamber Stage  15mm*15mm*15.5mm(effective distance) | 1  1 | Customized  Customized |
| 13 | Trigger | Pulse Pal (Gen2) | 2 | Sanworks |
| 14 | Arduino controller | Arduino UNO | 3 | Arduino |

**Supplementary Table 1 | List of parts used in the mLSFM setup.**


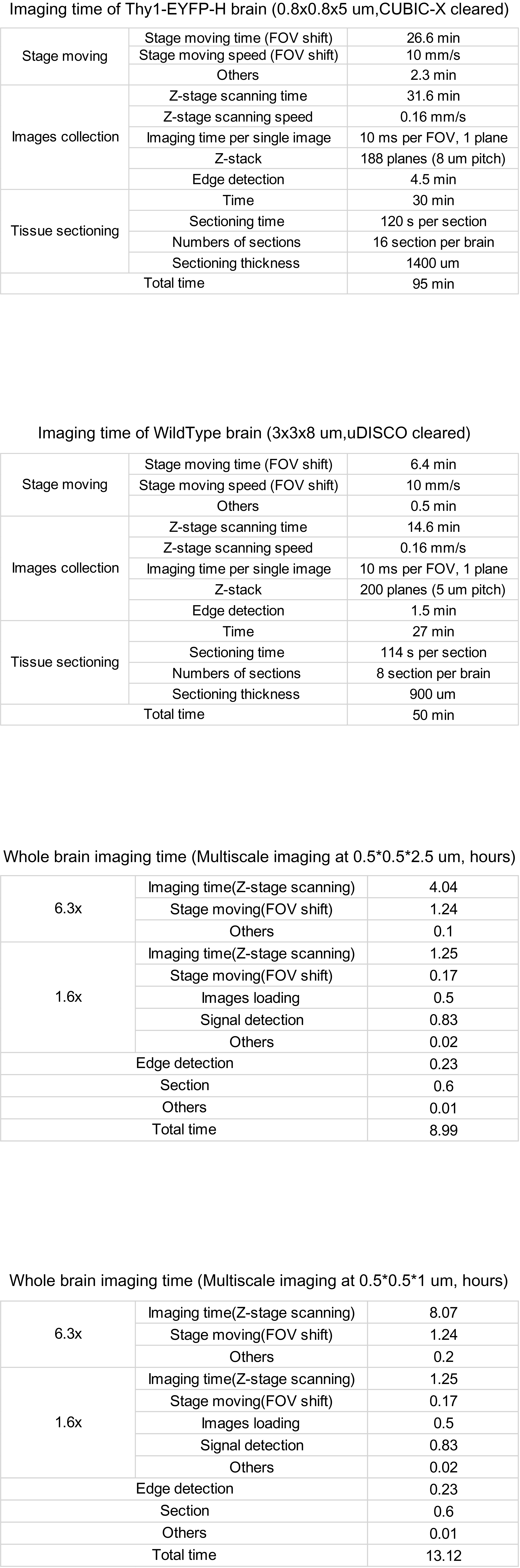


**Supplementary Table 2 | Analysis of imaging time at 0.8 x 0.8 x 5 μm^3^ spatial resolution for Thy1-EYFP-H mouse brains cleared using CUBIC-X protocol.** Specification of time for stage movement, tissue sectioning, and image processing.


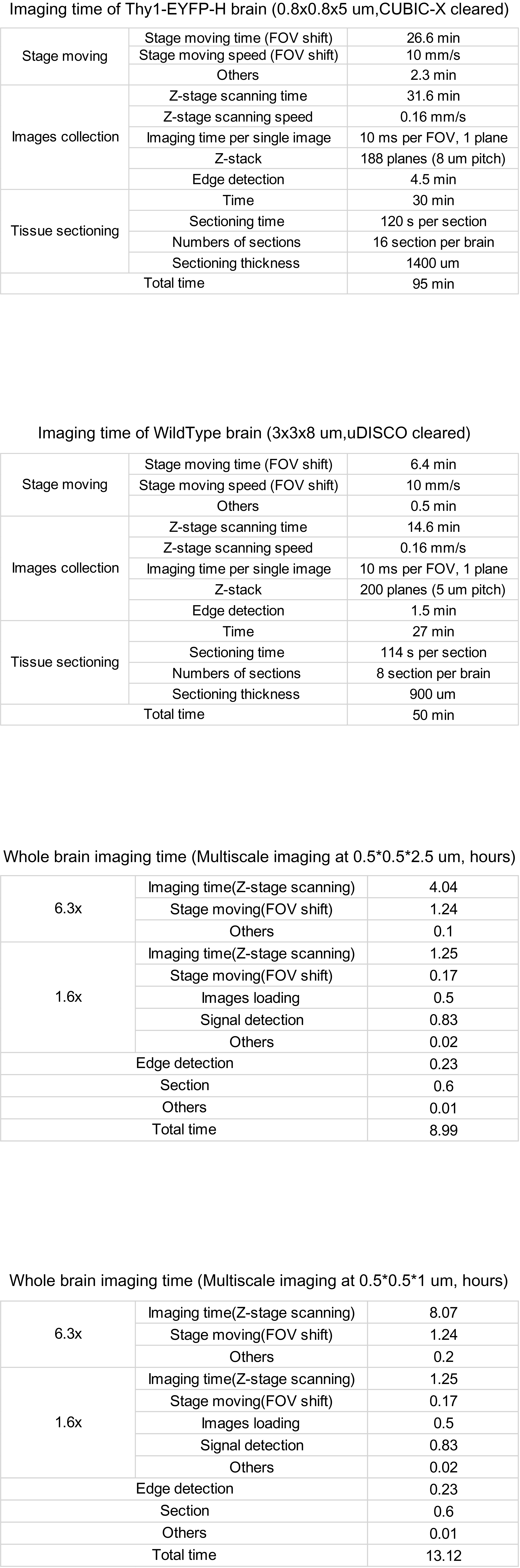


**Supplementary Table 3 | Analysis of imaging time at 3 x 3 x 8 μm^3^ resolution to reconstruct electrode tracks in uDISCO cleared brains.** Specification of time for stage movement, tissue sectioning, and image processing.


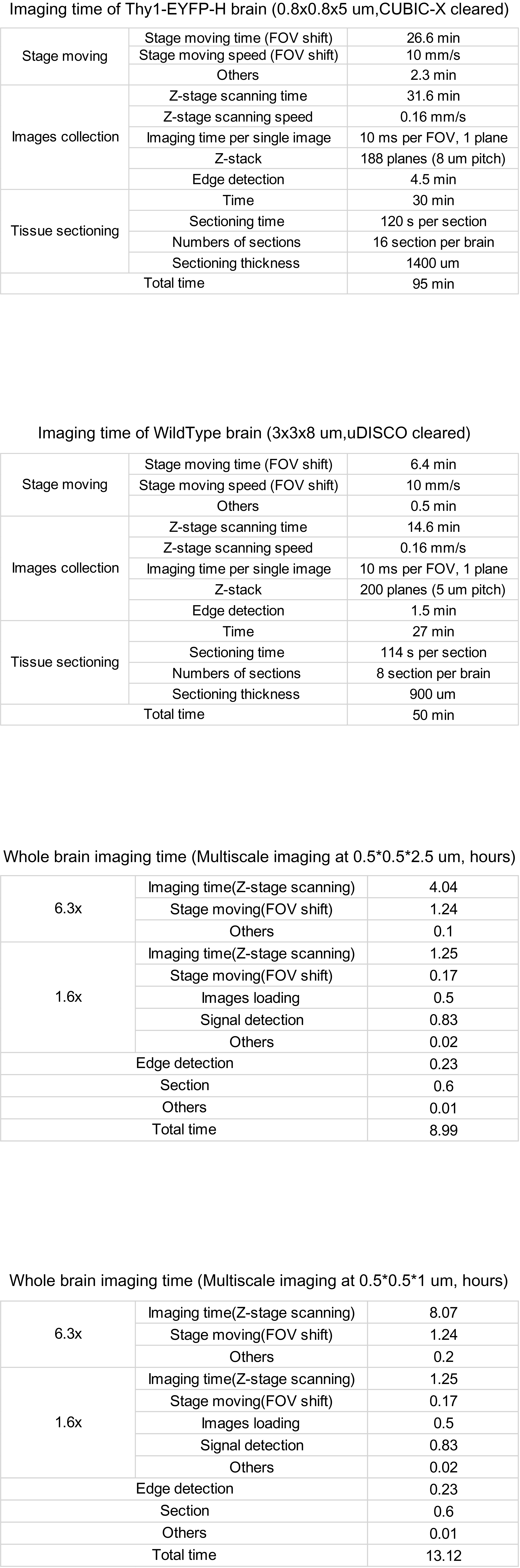


**Supplementary Table 4 | Analysis of multiscale imaging time at 0.3 x 0.3 x 2.5 μm^3^ for sparsely labeled neurons in mPFC.** Specification of time for stage movement, tissue sectioning, and image processing during multiscale imaging of CUBIC-X cleared brains.


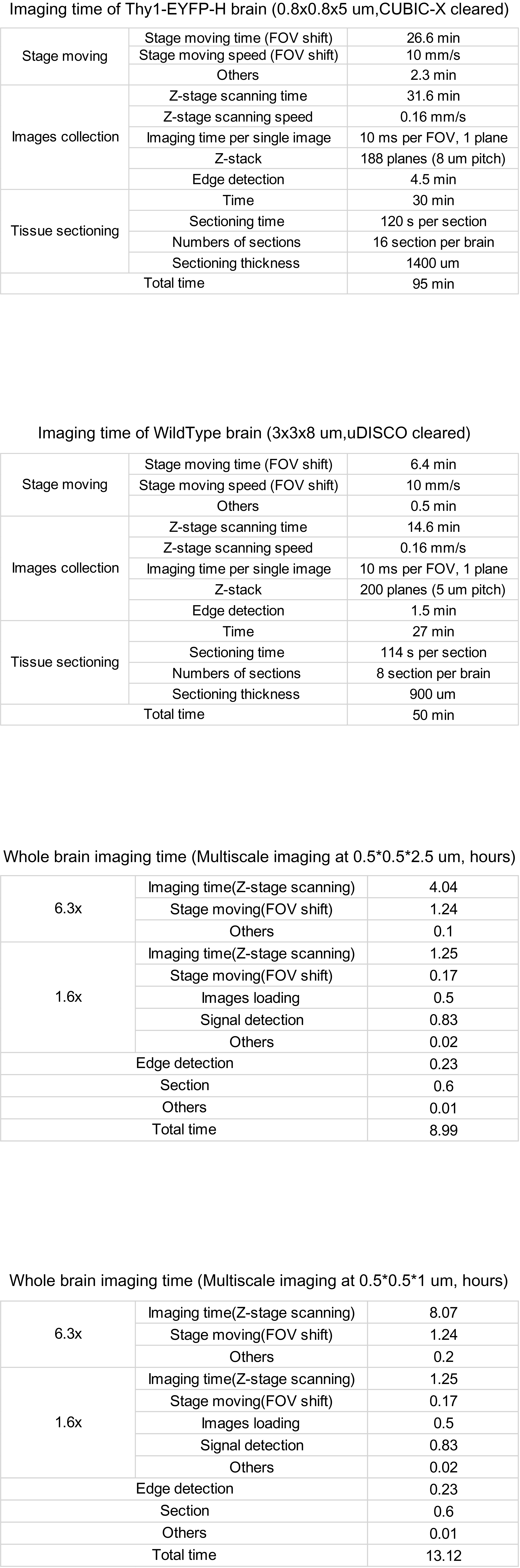


**Supplementary Table 5 | Analysis of multiscale imaging time at 0.3 x 0.3 x 1 μm^3^ for sparsely labeled neurons in mPFC.** Same as Table 4 but at 0.3 x 0.3 x 1 μm^3^ effective voxel size (after correction of tissue expansion).

**Supplementary Video 1 | Whole brain imaging of a Thy1-YFP-H transgenic mouse brain cleared using CUBIC-X protocol.**

**Supplementary Video 2 | Brain-wide imaging to reconstruct recording locations near thalamic reticular nucleus.**

**Supplementary Video 3 | Combination of single neuron morphologies in CCFv3.**
